# Synthesis and biological assessment of KojoTacrines as new agents for Alzheimer’s disease therapy

**DOI:** 10.1080/14756366.2018.1538136

**Published:** 2018-11-27

**Authors:** Youssef Dgachi, Hélène Martin, Rim Malek, Daniel Jun, Jana Janockova, Vendula Sepsova, Ondrej Soukup, Isabel Iriepa, Ignacio Moraleda, Emna Maalej, M. Carmo Carreiras, Bernard Refouvelet, Fakher Chabchoub, José Marco-Contelles, Lhassane Ismaili

**Affiliations:** a Laboratory of Applied Chemistry, Heterocycles, Lipids and Polymers, Faculty of Sciences of Sfax, University of Sfax, Sfax, Tunisia;; b Laboratoire de Chimie Organique et Thérapeutique, Neurosciences Intégratives et Cliniques EA 481, Univ. Bourgogne Franche-Comté, Besançon, France;; c Laboratoire de Toxicologie Cellulaire, Univ. Bourgogne Franche-Comté, Besançon, France;; d Department of Toxicology and Military Pharmacy, Faculty of Military Health Sciences, University of Defence, Hradec Kralove, Czech Republic;; e Biomedical Research Center, University Hospital Hradec Kralove, Hradec Kralove, Czech Republic;; f Department of Organic Chemistry and Inorganic Chemistry, School of Biology, Environmental Sciences and Chemistry, University of Alcalá, Alcalá de Henares, Spain;; g Laboratoire Matériaux, Traitement et Analyse (LMTA), Institut National de Recherche et d'Analyse Physico-chimique Technopole, Ariana-Tunis, Tunisia;; h Research Institute for Medicines (iMed.ULisboa), Faculty of Pharmacy, Universidade de Lisboa, Lisboa, Portugal;; i Laboratory of Medicinal Chemistry, IQOG, CSIC, Madrid, Spain

**Keywords:** Alzheimer disease, kojic acid, multitarget small molecules, tacrine

## Abstract

In view of the multifactorial nature of Alzheimer’s disease (AD), multitarget small molecules (MTSM) represent the most potent and attractive therapeutic strategy to design new drugs for Alzheimer’s disease therapy. The new MTSM KojoTacrines (KTs) were designed and synthesized by juxtaposition of selected pharmacophoric motifs from kojic acid and tacrine. Among them, 11-amino-2-(hydroxymethyl)-12-(3-methoxyphenyl)-7,9,10,12-tetrahydropyrano [2',3':5,6] pyrano[2,3-*b*]quinolin-4(8*H*)-one (**KT2d**) was identified as less-hepatotoxic than tacrine, at higher concentration, a moderate, but selective human acetylcholinesterase inhibitor (IC_50_ = 4.52 ± 0.24 µM), as well as an antioxidant agent (TE = 4.79) showing significant neuroprotection against A*β*_1–40_ at 3 µM and 10 µM concentrations. Consequently, **KT2d** is a potential new hit-ligand for AD therapy for further biological exploration.

## Graphical Abstract

**Figure:**
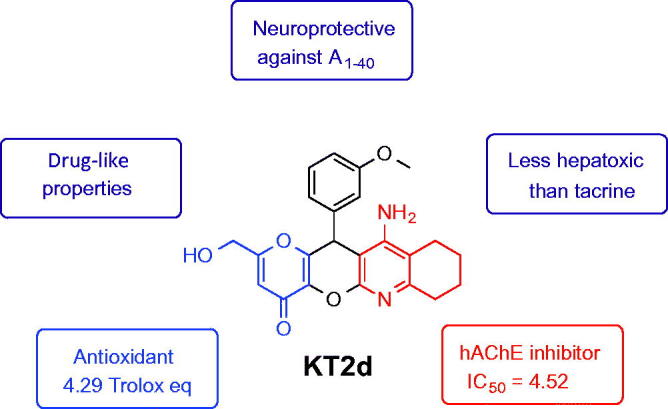

## Introduction

The World Health Organization claims that 46 million people worldwide suffer from Alzheimer’s disease (AD). It is likely that this number may significantly rise as the life expectancy increases^1^ ^,^ ^2^. AD is a multifactorial neurodegenerative disorder, inducing a progressive memory loss, decline in language skills, and other cognitive impairments^3^ ^,^ ^4^. Although the aetiology of the disease is still not fully understood^5^, low levels of acetylcholine (ACh), the aggregation of amyloid-beta peptide (A*β*), hyperphosphorylation of tau protein, oxidative stress, and accumulation of biometals (Cu, Fe, Zn) are thought to play key roles in the pathophysiology, progress and development of AD^6^ ^,^ ^7^. AD patients are currently treated either with the *N*-methyl-D-aspartate receptor antagonist, memantine^8^, or donepezil, rivastigmine, and galantamine^9^, three acetylcholinesterase inhibitors (AChEIs), but with limited therapeutic success. Consequently, there is an urgent need for new and more efficient drugs for AD therapy.

Because of the complex nature of AD, multitarget small molecules (MTSM), able to interact simultaneously with the different enzymatic systems or receptors involved in the pathology, have emerged as one of the most promising therapeutic strategies in order to design and identify new drugs for AD^10–15^. Following this paradigm, we have prepared a number of new MTSM by multicomponent reactions (MCR)^16–19^ as AChEIs, showing neuroprotective effect and strong antioxidant activity.

In this article, we report the synthesis and biological evaluation of novel MTSM, called KojoTacrines (KTs), resulting from the combination of selected motifs present in kojic acid (KA) and tacrine (Figure 1). Tacrine was the first marketed and approved AChEI for AD therapy although discontinued shortly after its approval due to its hepatotoxicity^20^. Despite this, tacrine has been largely used as a template to design new non-hepatotoxic analogues for AD^13^ ^,^ ^15^. Our current choice for tacrine to build the new anti-Alzheimer agents is based on the fact that (1) tacrine derivatives are easily available by simple synthetic protocols; (2) tacrine is a potent ChE inhibitor, able thus to increase the level of neurotransmitter ACh in the brain, and consequently, it has been assumed that (new) tacrine derivatives should behave similarly with particular effects depending on their structure; (3) we have demonstrated that it is possible to design and prepare non-hepatotoxic tacrine derivatives showing ChEs inhibition^21^; and (4) last but not least, the number of published papers dealing with tacrine derivatives as anti-Alzheimer should be also considered as a strong point in order to support our choice. So, the purpose of this preliminary communication is to identify a new tacrine-based scaffold and its derivatives. KA, a naturally occurring fungal metabolite, is a recognized antioxidant agent^22^ ^,^ ^23^. Thus, considering KA ability to scavenge reactive oxygen species, 12 new kojo tacrines (KTs) were designed, synthesized, and evaluated as potential cholinesterase inhibitors (ChEIs) enhanced with antioxidant properties. As a result, 11-amino-2-(hydroxymethyl)-12–(3-methoxyphenyl)-7,9,10,12-tetrahydropyrano[2', 3':5,6]pyrano[2,3-*b*]quinolin-4(8*H*)-one (**KT2d**) was identified as a hit-agent, less hepatotoxic than tacrine, completely selective against AChE, showing potent antioxidant properties and displaying a very significant neuroprotection against amyloid beta (A*β*).

**Figure:**
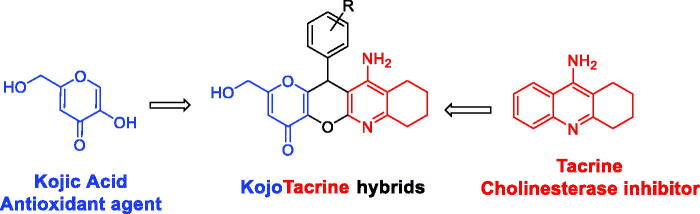
**Figure 1**. Design of KojoTacrine hybrids.

## Methods

### Chemistry

The synthetic method of only compound **KT2d** is described here, for the rest of compounds readers are invited to refer to Supporting Information section.

#### Amino-2-(hydroxymethyl)-12–(3-methoxyphenyl)-7,9,10,12-tetra hydropyrano[2',3':5,6] pyrano[2,3-b]quinolin-4(8H)-one (2d)

A solution of previously described compound **1d** ^24^ (0.5 g, 1.5 mmol) in 1,4-dioxane (30 ml) with cyclohexanone (0.23 g, 2.25 mmol) and AlCl_3_ (0.31 g, 2.25 mmol) was heated at 110 °C for 2 h and monitored by TLC (CH_2_Cl_2_/MeOH, 8/2, v/v). When the reaction was complete, the reaction mixture was diluted with a solution of CH_2_Cl_2_/water (1/1) and treated with an aqueous solution of sodium hydroxide (10%) until pH 11–12. The mixture was stirred for 20 min and then extracted with CH_2_Cl_2_, dried over anhydrous sodium sulfate, filtered, and the solvent was evaporated to generated the desired product **2d** (0.36 g, 59%): mp >260 °C; IR (KBr) ν 3470 (OH), 3330 and 3250 (NH_2_), 1725 (C = O) cm^−1^; ^1^H NMR (DMSO-d_6_, 400 MHz) δ 1.72 (m, 4H), 2.18–2.22 (m, 1H), 2.33–2.37 (m, 1H), 2.55–2.66 (m, 2H), 3.72 (s, 3H, OCH_3_), 4.20–4.28 (m, 2H), 5.32 (s, 1H), 5.69 (br s, 2H, NH_2_), 6.32 (s, 1H), 6.77–6.40 (m, 2H_arom_), 6.97 (s, 1H_arom_), 7.21–7.25 (m, 1H_arom_); ^13^C NMR (DMSO-d_6_, 100 MHz) δ 22.4 (CH_2_), 22.7 (CH_2_), 23.4 (CH_2_), 32.5 (CH_2_), 40.6 (CH), 55.5 (OCH_3_), 59.8 (CH_2_), 96.7 (C), 111.7 (CH), 112.7 (CH), 112.8 (C), 114.9 (CH), 120.2 (CH), 130.4 (CH), 137.8 (C), 142.6 (C), 150.0 (C), 152.1 (C), 153.7 (C), 154.3 (C), 159.7 (C), 168.1 (C), 171.04 (C). Anal. Calcd for C_23_H_22_N_2_O_5_: C, 67.97; H, 5.46; N, 6.89. Found: C, 68.21; H, 5.38; N, 7.01.

### Biological evaluation

The methods for the *in vitro* cytotoxicity in HepG2 cells, the inhibition of *Ee*AChE, eqBuChE, and hChE, the neuroprotective assay, as well as the ORAC assay have been briefly described

#### In vitro cytotoxicity of KojoTacrines in HepG2 cells^*17*^ ^,^ ^*18*^ ^,^ ^*25*^

The culture of HepG2 cells were incubated at 37 °C in a humidified atmosphere of 5% CO_2_ and 95% air. The culture medium was refreshed after 24 h of incubation and 100 µl of six different concentrations (1–1000 µM) of test compounds or DMSO (0.1%) were then added. The cell viability was monitored by colourimetric assay with 3-[4,5-dimethylthiazol-2-yl]-2,5-diphenyl-tetrazolium bromide (MTT) ^26^.

#### Inhibition of EeAChE and eqBuChE of compounds KTs 2a–d, 2f–h, and 2l^*17*^ ^,^ ^*27*^

Ellman’s protocol was followed to perform the anticholinesterasic activity^28^. Briefly, the reaction was carried out in a final volume of 3 ml of a phosphate-buffered solution (0.1 M) at pH =8.0 containing (2.625 µl, 0.35 mM) of 5,5’-dithiobis-2-nitrobenzoic acid (DTNB), 40 µl 1% w/v Bovine Albumin Serum phosphate-buffered solution (BSA), (29 µl, 0.035 U/ml) of *Ee*AChE or (60 µl, 0.05 U/ml) of eqBuChE, and 3 µl of tested compound, which were incubated for 10 min. Then, the acetylthiocholine iodide or butyrylthiocholine was added, and the reaction was incubated for additional 15 min. The activity in absence of compound was used as control. The absorbances were measured in a spectrometric plate reader at 412 nm and the IC_50_ were determined using GraphPad Prism 5.

#### Inhibition of human cholinesterases^*17*^ ^,^ ^*27*^

Inhibitory activities of the tested compounds were expressed as IC_50_, i.e., concentration of compound required for 50% reduction in cholinesterase activity using Ellman’s method^28^. The assay was carried out in medium (100 µl) containing 10 µl of cholinesterase obtained from human erythrocyte hemolyzate, out of blood samples, 40 µl of 0.1 M PBS (pH 7.4), 20 µl of 0.01 M DTNB, 10 µl of different concentrations of tested compound (10^−3^–10^−9 ^M). This blend was preincubated for 5 min and 20 µl of 0.01 M substrate (ATCh or BTCh) was added to initiate the reaction. The activity was determined by measuring the increase in absorbance at 412 nm at 37 °C in 2 min intervals using Multi-mode microplate reader Synergy 2 (Vermont, USA). The IC_50_ values are expressed as a mean ± SEM.

#### Neuroprotection assay^*29*^

SH-SY5Y cell culture and treatment with compounds **2b–d** and **2f**. The cultures of human SH-SY5Y neuroblastoma cells kept under a CO_2_/air (5%/95%) humidified atmosphere at 37 °C for 24 h were treated with 100 µl of the test compounds or DMSO (0.1%) in DMEM/F12 (1:1) medium supplemented with 1% fetal bovine serum, 1X non-essential amino acids, 100 units/ml penicillin and 10 mg/ml streptomycin. Treatment medium was renewed every day for up to 48 h of treatment. MTT assay was then performed.

#### Effect of 2b–d and 2f on O/R, Αβ_1–40_ peptide-induced cell death in SH-SY5Y cells

SH-SY5Y cells were seeded in 96-well culture plates at a density of 8 × 10^4^ cells per well in DMEM/F12 (1:1) medium supplemented with 10% fetal bovine serum, 1X non-essential amino acids, 100 units/mL penicillin and 10 mg/mL streptomycin (Dutscher, France). After 24 h of incubation, the cultures were treated with 100 µl of the test compounds or DMSO (0.1%) in the same medium. Following 24 h, the cells were co-incubated with oligomycin A (10 µM)/rotenone (30 µM) (Sigma, France) or A*β*_1–40_ (30 µM) with or without the tested compounds **2b–d** and **2f** at nontoxic concentrations against cells for an additional period of 24 h. MTT assay was then performed to determine the % of cell viability.

#### Oxygen radical absorbance capacity assay^*30*^ ^,^ ^*31*^

The antioxidant activity of KTs was determined using the ORAC-FL method described by Dávalos et al.^30^ using fluorescein as a fluorescent probe with minor modification. Briefly, fluorescein and antioxidant were incubated in a black 96-well microplate (Nunc) for 15 min at 37 °C. The 2,2’-azobis(amidinopropane) dihydrochloride (AAPH) was then added quickly using the built-in injector of Varioskan Flash plate reader (Thermo Scientific). The fluorescence was measured at 485 nm (excitation wavelength) and 535 nm (emission wavelength) every min for 60 min.

### Molecular modelling

#### Molecular modelling of compound KT2d

For docking experiments, Autodock Vina^32^ software was employed. Compounds (*R*)-**KT2d** and (*S*)-**KT2d** were prepared with Discovery Studio, version 2.1, software package, using standard bond lengths and bond angles. With the CHARMm force field^33^ and partial atomic charges, the molecular geometries of the compounds were energy-minimized using the adopted-based Newton–Rapson algorithm until the rms gradient was below 0.01 kcal (mol-Å)^−1^. Three-dimensional crystal structure of hAChE complexed with fasciculin-II (PDB: 1B41) was retrieved from the Protein Data Bank (PDB). Before docking the ligands into the protein, this was prepared by removing all water molecules, heteroatoms, any co-crystallized solvent, and the ligand. Protein model tool in Discovery Studio, version 2.1, software package was used to assign proper bonds, bond orders, hybridization, and charges. CHARMm force field was applied using the receptor-ligand interactions tool in Discovery Studio, version 2.1, software package. AutoDockTools (ADT; version 1.5.4) was used to add hydrogens and partial charges for proteins and ligands using Gasteiger charges. Selected side chains into the target macromolecule are allowed to change their conformations at the same time as the ligand that is being docked. Using the AutoTors module, the macromolecule side chains chosen to be flexible are Trp286, Tyr124, Tyr337, Tyr72, Asp74, Thr75, Trp86 and Tyr341. The docking box was displayed using ADT and it is big enough to include whole protein target (“blind docking”). A grid box of 60 × 60 × 72 with grid point spacing of 1 Ǻ was positioned at the middle of the protein (*x* = 116.546; *y* = 110.33; *z* = −134.181). Default parameters were used except num_modes, which was set to 40. The AutoDock Vina docking procedure used was previously validated^25^. The scoring function of AutoDock Vina was chosen, and the docking poses for each ligand were analyzed by examining their relative total energy score. The more energetically favorable conformation was selected as the best pose. The structures of the macromolecule and ligands, as well as the docking results, were processed using Discovery Studio software.

## Results and discussion

### Chemistry

**Scheme 1.**
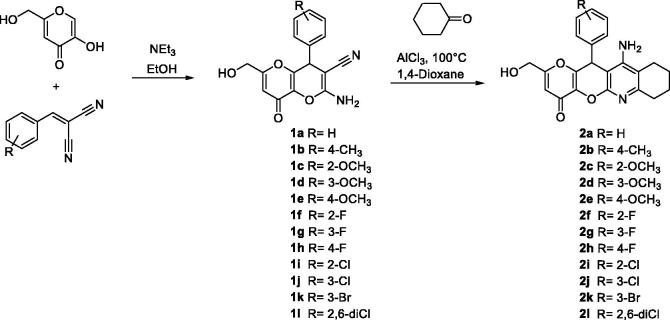
Synthesis of racemic KojoTacrines **2a**-**l**

The synthesis of racemic KTs 11-amino-2-(hydroxymethyl)-12-aryl-7,9,10,12-tetrahydropyrano[2',3':5,6]pyrano[2,3-*b*]quinolin-4(8*H*)-ones (**2a**–**l**) was carried out in two steps by using easily available precursors, as shown in Scheme 1. Thus, KA was reacted with selected arylidenemalonitriles, in the presence of catalytic amounts of triethylamine, in ethanol, at 80 °C, for 5 min, to give the known 2-amino-6-(hydroxymethyl)-8-oxo-4-phenyl-4,8-dihydropyrano[3,2-*b*]pyran-3-carbonitriles **1a**–**l** in very good yields^34^. Next, Friedländer-type reaction^35^ of compounds **1a–l** with cyclohexanone, under the usual experimental conditions^35^, afforded the expected racemic KTs **2a–l** (Scheme 1).

All new KT ligands showed analytical and spectroscopic data in good agreement with their structures (see **Experimental Part**).

### Biological evaluation

#### *In vitro* cytotoxicity of KTs 2a–l in HepG2 cells

Hepatotoxicity is the most critical biological concern when dealing with new tacrine derivatives. In this sense, all new KTs were submitted to the *in vitro* cell viability test (MTT assay) using human hepatocellular carcinoma cell line (HepG2)^26^, as the most accepted probe to evaluate hepatotoxic effects. For this experiment, six different concentrations from 1 to 1000 µM were used. As shown in Table 1, it is important to note that no significant toxicity at concentrations up to 1000 µM was observed for **KT2b**; therefore, this compound can be considered as non-hepatotoxic. As shown, tacrine and KTs **2e**, **2i–l** start to exert a marked cytotoxicity at 300 µM, in contrast to KTs **2a–d** and **2f–h**, which showed low toxicity at this concentration; however, at 1000 µM, these KTs induce cell death but not as much as tacrine, remaining from 5- to 7-fold less toxic than tacrine.

**Table 1.** Effects of KojoTacrines **2a-l** on human hepatocyte HepG2 cell viability.^a^

	1 µM	3 µM	10 µM	30 µM	100 µM	300 µM	1000 µM
Tacrine	112.2 ± 4.0	113.6 ± 4.8	107.8 ± 3.4	109.6 ± 4.2	101.9 ± 2.4	42.4 ± 2.0***	10.9 ± 2.0***
2a	96.8 ± 1.0	94.9 ± 2.9	99.3 ± 1.7	105.2 ± 5.3	108.1 ± 5.0	95.0 ± 3.4	50.3 ± 5.7**
2b	105.1 ± 3.1	109.5 ± 3.2	105.4 ± 1.9	107.0 ± 2.9	112.9 ± 8.4	108.5 ± 5.7	87.9 ± 5.2
2c	94.7 ± 2.4	103.4 ± 2.8	99.0 ± 1.1	98.2 ± 2.2	96.1 ± 1.5	88.5 ± 7.1	70.7 ± 7.7*
2d	100.8 ± 2.5	98.0 ± 4.1	101.5 ± 3.2	101.7 ± 1.8	99.0 ± 1.1	87.2 ± 6.1	65.5 ± 1.3*
2e	95.5 ± 5.5	96.1 ± 7.1	95.9 ± 5.6	98.3 ± 3.8	86.1 ± 3.8	70.9 ± 4.5*	41.7 ± 3.0***
2f	108.0 ± 6.2	96.1 ± 2.5	99.2 ± 2.8	102.2 ± 7.1	100.9 ± 9.2	95.3 ± 4.1	64.0 ± 8.0*
2g	101.2 ± 7.1	96.5 ± 7.4	99.1 ± 3.8	108.8 ± 6.1	103.1 ± 6.4	90.5 ± 9.6	62.6 ± 9.7*
2h	97.4 ± 2.3	95.9 ± 3.8	100.5 ± 1.5	101.4 ± 0.6	102.2 ± 1.8	87.2 ± 6.7	55.3 ± 6.0**
2i	98.6 ± 1.4	100.1 ± 1.9	99.4 ± 1.2	104.7 ± 2.6	95.2 ± 4.7	64.4 ± 6.3*	23.1 ± 6.0***
2j	97.6 ± 2.0	101.4 ± 5.4	105.1 ± 5.5	99.6 ± 2.4	102.1 ± 6.9	52.7 ± 8.3**	14.5 ± 6.4***
2k	101.4 ± 4.8	104.0 ± 8.9	94.9 ± 2.6	95.8 ± 2.5	100.7 ± 3.1	60.1 ± 10.4*	37.2 ± 5.9***
2l	99.9 ± 2.7	102.1 ± 2.3	102.2 ± 2.6	99.9 ± 3.2	88.9 ± 8.0	68.3 ± 9.3*	50.5 ± 7.5**

a Data are expressed as percent of cell viability as compared to control cultures (DMSO-treated cells). Means ± SEM of triplicates from three different cultures. *p < 0.05, **p < 0.01, ***p < 0.001, as compared to the control cultures (one-way ANOVA).

Based on the hepatotoxicity results, the next step was to investigate the capacity KTs **2a–d**, **2f–h**, and **2l**, showing values >50% in the HepG2 cell viability test, for their ChE inhibition capacity and ORAC power.

#### Evaluation of AChE and BuChE inhibition of KTs 2a–d, 2f–h, and 2l

The inhibition of *Electrophorus electricus* AChE (*Ee*AChE) and serum horse BuChE (eqBuChE) was carried out following Ellman’s assay^28^, using the synthesized KTs and tacrine, as a positive control for comparative purposes.

As shown in Table 2, all KTs investigated are less potent ChEIs than tacrine. However, they are moderate, but selective AChEIs, in the micromolar range, showing IC_50_ (µM) values from 0.64 ± 0.06 (**2d**) to 3.44 ± 0.04 (**2h**). The most potent *Ee*AChEIs was ligands **2d** with IC_50_ values equal to 0.64, being only approximately 20-fold less active than tacrine (IC_50_ = 0.031 µM). For the inhibition of eqBuChE, the KTs showed micromolar inhibition ranging from 4.54 to 12.15 µM. The most potent was KT **2 g** (IC_50_ = 4.54 µM). Regarding the structure-activity relationships, no evident and clear trends could be observed for the inhibition of *Ee*AChE and eqBuChE.

**Table 2.** Inhibition of *Ee*AChE, hACHE, eqBuChE, (IC_50_, μM)^a^, and ORAC-FL values for KTs **2a-d, 2f-h, 2l**, tacrine, and ferulic acid (FA). 
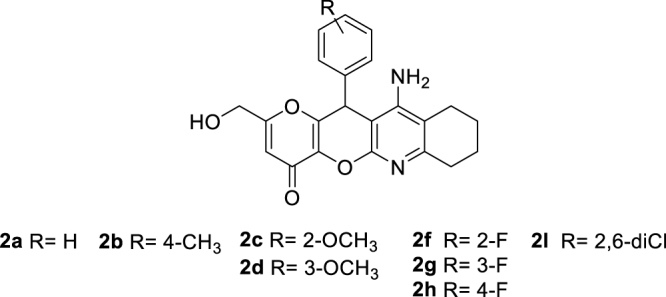

KT	R	*Ee*AChE (IC_50_, μM)	*eq*BuChE (IC_50_, μM)	hAChE (IC_50_ µM)	ORAC
**2a**	H	3.30 ± 0.09	12.15 ± 0.09	–b	2.58 ± 0.22
**2b**	4-CH_3_	1.40 ± 0.00	10.09 ± 0.09	13.7 ± 1.7	2.96 ± 0.18
**2c**	2-OCH_3_	2.39 ± 0.02	–b	–c	6.05 ± 0.41
**2d**	**3-OCH_3_**	**0.64 ± 0.06**	**–**b	4.52 ± 0.24	**4.79 ± 0.39**
**2f**	2-F	2.11 ± 0.01	6.69 ± 0.10	–b	3.85 ± 0.42
**2g**	3-F	2.14 ± 0.12	4.54 ± 0.20	–c	4.34 ± 0.17
**2h**	4-F	3.44 ± 0.04	–b	–c	4.69 ± 0.18
**2l**	2,6-diCl	2.12 ± 0.09	–b	–c	6.14 ± 0.40
Tacrine	–	0.031 ± 0.006	0.005 ± 0.001	0.13 ± 0.00	0.2 ± 0.1
FA	–	–c	–c	–c	3.74 ± 0.22
KA	–	–c	–c	–c	2.51 ± 0.17

a Inhibition curves were obtained by nonlinear regression. Each IC_50_ value is the mean ± SEM of quadruplicate of at least three different experiments.

^b^% Inhibition under 50% at 10 μM.

^c^Not determined.

Next, the most balanced KTs **2b–d** and **2f**, based on the hepatotoxicity data and inhibitory activities against *Ee*AChE and eqBuChE, were evaluated for their ability to inhibit human AChE (hAChE) and human BuChE (hBuChE). Regarding hBuChE the four products showed a percentage of inhibition under 50% at 10 µM and, consequently, their IC_50_ values were not determined. However, for hAChE, compound KT**2d** showed significant inhibition (IC_50_ = 4.52 µM), whereas the non-hepatotoxic KT**2b** showed a lower inhibitory activity equal to 13.7 µM, as it is shown in Table 2. At this point of our project, KT **2d** had been identified as a promising compound on diverse biological tests, in spite that this ligand shows lower AChE inhibition than tacrine, because its hepatotoxic effects are lower, still showing a quite good, in the low micromolar range, and selective, AChE inhibition. A very finely balanced situation has been found. Next, as planned at the beginning of the project, the analysis of the antioxidant capacity of **KT 2a–d**, **2f–h**, and **2l** was carried out.

#### Evaluation of antioxidant power of KTs 2a–d, 2f–h, and 2l

The antioxidant ability of KTs **2a–d**, **2f–h**, and **2l** was then evaluated using the Oxygen Radical Capacity (ORAC-FL) method^30^ ^,^ ^36^ with tacrine and ferulic acid (FA)^29^ ^,^ ^37^ as negative and positive controls, respectively. Results are expressed in the Trolox equivalents (TE) unit in relation to radical scavenging properties of Trolox. As shown in Table 2, all evaluated KTs exhibited good radical scavenging properties, the ORAC values ranging from 2.58 TE (**2a**) to 6.14 TE (**2l**), comparing favourably with KA (2.51 TE) and ferulic acid (FA) (3.74 TE). The most potent antioxidant KTs were those bearing a methoxy group (**2c**) in position C2, and two chloro atoms in positions C2 and C6 (**2l**) showing 6.05 and 6.14 TE, respectively. Interestingly, the most balanced KT**2d**, regarding its hepatotoxicity, neuroprotection, cytotoxicity, and ChE inhibition, showed a very high 4.79 TE, 1.3-fold more potent than FA. Based on these interesting results, next, we decided to investigate the neuroprotective properties of some selected non-hepatotoxic KTs.

#### Neuroprotective capacity of KojoTacrines 2b–d and 2f

The ability to prevent the human neuroblastoma cell line SH-SY5Y cell death induced by toxic insults such as Α*β*_1–40_, implicated in ROS production and apoptosis-related signalling pathways^38^, and oligomycin/rotenone (O/R)^39^, was then assayed to determine the neuroprotective capacity of the four less hepatotoxic KTs **2b–d** and **2f** at nontoxic concentration against SH-SY5Y 3 and 10 µM, using melatonin and KA as reference molecules. For the evaluation of the neuroprotective effect, the cytotoxicity of O/R (10 µM/30 µM) on SH-SY5Y cell viability was determined as well as the toxicity of the selected KTs **2b–d** and **2f** at. As shown in Table 3, only KT**2d** showed a significant and similar neuroprotection against A*β*_1–40_ insult at 3 and 10 µM and, therefore, slightly less active than KA. It must also be recognized that the non-hepatotoxic KT**2b** showed very poor neuroprotective activity on any of the toxic insults investigated.

**Table 3.** Neuroprotective activity of KTs **2b-d** and **2f** on Oligomycin/Rotenone (O at 10 µM)/Rotenone (R at 30 µM), and A*β*_1–40_ (30 µM) peptide-induced cell death in SH-SY5Y cells.

KojoTacrine	Dose	O/R (%)	A*β*_1–40_ (%)
**2b**	3 µM	19.66 ± 0.07	27.85 ± 0.15*
** **	10 µM	3.65 ± 0.07	np
**2c**	3 µM	12.18 ± 0.08	np
** **	10 µM	16.56 ± 0.02	np
**2d**	3 µM	24.35 ± 0.05*	42.92 ± 0.20***
** **	10 µM	12.53 ± 0.09	41.19 ± 0.18***
**2f**	3 µM	16.50 ± 0.07	25.10 ± 0.06*
** **	10 µM	np	4.1 ± 0.04
**Melatonin^17^**	1 µM	50.1 ± 3.42**	35.5 ± 4.1**
	3 µM	59.1 ± 4.50**	60.2 ± 3.57***
**Kojic acid**	1 µM	np	51.56 ± 0.18 ***
	3 µM	np	65.11 ± 0.20***

Neuroprotection was expressed in percentage viable cells of control condition. Data are the means ± SEM of quadruplicates from three different cultures; *p < 0.05, **P ≤ 0.01,***p < 0.001, as compared to the control cultures (one-way ANOVA); np: not protective.

Finally, based on the results described above, KT**2d** was selected as our hit-compound. Thus, its docking analysis was carried out, using the program Autodock Vina^32^ and the crystal structure (PDB: 1B41) of hAChE available as a complex with fasciculin II, in order to justify its binding properties on hAChE.

#### Molecular modelling of compound KT2d

Since compound **KT2d** has a chiral centre at the pyran ring, the reported biological data refer to the racemic mixture, and the observed whole result is a combination of the effects of both enantiomers. Nevertheless, to obtain more information about the binding properties, docking studies were performed on both (*R*)- and (*S*)-enantiomers of compound **KT2d**. The docking procedure was applied to the whole protein target, without imposing the binding site (“blind docking”). Flexible docking experiments have been performed for (*R*)- and (*S*)-enantiomers of **KT2d**. To address receptor flexibility in hAChE, the rearrangement of the side chains of eight residues, Trp286, Tyr124, Tyr337, Tyr72, Asp74, Thr75, Trp86, and Tyr341 was allowed. The movement of these residues may increase the size of the gorge and thus facilitate the access of bulky ligands to the catalytic site^25^ ^,^ ^40^.

**Figure 2.**
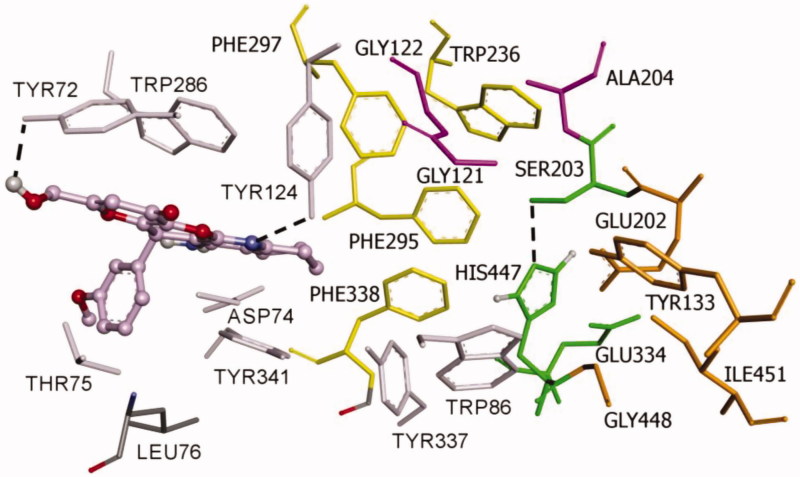
**Binding mode of inhibitor (*R*)-KT2d at the active site of hAChE.** Compound **(*R*)-KT2d** is pictured as ball and sticks in pink and the side chains of the mobile residues are represented in the pale pink. The sub-sites of the active site were coloured: oxyanion hole (OH) in fuchsia, anionic sub-site (AS) in orange, except Trp86, acyl binding pocket (ABP) in yellow, catalytic triad (CT) in green and PAS in light violet. Hydrogen bond interactions are represented as dashed black lines.

**Figure 3.**
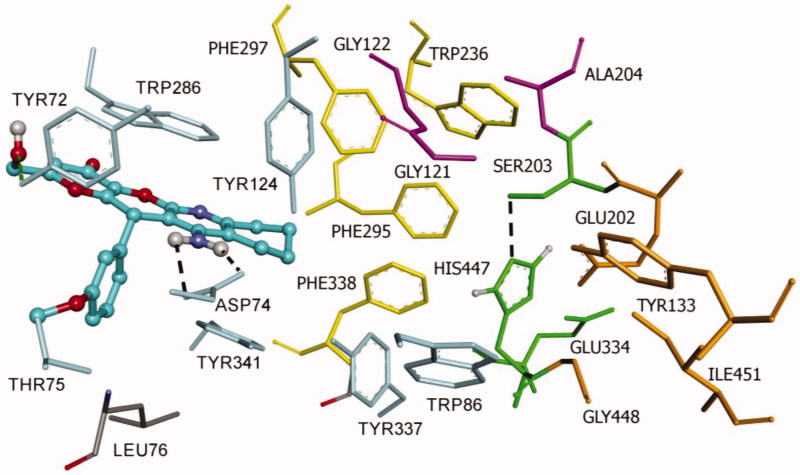
**Docking pose of inhibitor (*S*)-KT2d at the active site of hAChE.** Compound **(*S*)-KT2d** is illustrated as ball and sticks in blue and the side chains of the mobile residues are represented in pale blue. The subsites of the active site were coloured: Oxyanion hole (OH) in fuchsia, anionic sub-site (AS) in orange, except Trp86, acyl binding pocket (ABP) in yellow, catalytic triad (CT) in green, and peripheral anionic subsite (PAS) in light violet. Hydrogen bond interactions are represented as dashed black lines.

Both (*R*)- and (*S*)-enantiomers of compound **KT2d** were studied in docking simulations. The binding energy of (*S*)-enantiomer (−10.4 Kcal/mol) was lower than that of the (*R*)-enantiomer (−9.6 Kcal/mol). As depicted in Figures 2 and 3, both enantiomers are positioned in the enzyme active site in such way to interact with the aminoacids at the rim of the cavity and act as a barrier preventing the entry of the substrate into the active site. The fused-rings, part of compound **KT2d**, which has an approximately planar structure, make π–π-stacking with the aromatic aminoacids at the rim of the peripherial active site (PAS), including Trp286 and Tyr341. The phenyl ring showed π-alkyl hydrophobic interaction with Leu76 (Figures S1 and S2, see Supporting Information).

Furthermore, the binding is also supported by the formation of hydrogen bonds. For compound (*R*)-**KT2d,** a hydrogen bond between the Tyr124-OH and the nitrogen atom of the pyranotacrine moiety is established (Figure 2). For compound (*S*)-**KT2d**, the amino group established hydrogen bond interactions with Asp74 carboxylate group (key residue of PAS) and the oxygen atom of the methoxy group establishes a hydrogen bond interaction with the backbone-NH-group of Thr75 (Figure 3). Hydrogen bonding interactions with residue Tyr72 were observed for both enantiomers. As can be seen in Figure 5, another π-anion interaction has been established for (*S*)-**KT2d** enantiomer between the phenyl moiety and the Asp74.

On the basis of the set of interactions, which were identified between the (*R*)-enantiomer [Figure 2 and Figure S1 (Supporting Information)], the (*S*)-enantiomer [Figure 3 and Figure S2 (Supporting Information)] and the protein residues within the active site gorge of both enzymes, we conclude that the OH group and the phenyl ring are likely to be important features for these derivatives to exhibit hAChE inhibitory activities.

#### ADME of compounds KT2a–l

Theoretical calculations of the ADME (Absorption, Distribution, Metabolism, and Excretion) properties of both enantiomers of tacrine derivatives **KT2a–l** were accomplished using the QikProp module of Schrodinger suite (QikProp, version 3.8, Schrodinger, LLC, New York, NY, 2013) running in normal mode, for assessing the druggability. Drug kinetics and exposure of tissues to drug influences the pharmacological activity and the performance of a drug, which is ultimately determined by its ADME properties. Nearly 45 physically significant descriptors and pharmacologically relevant properties were predicted and investigated (Table S1, see Supporting Information).

In particular, for compound **KT2d**, the more significant observed data were the following: total Solvent Accessible Surface Area, in square angstroms, using a probe with a 1.4 Å radius (**SASA = 653.798** and **670.621** for (*R*)- and (*S*)-enantiomer, respectively; limits 300.0–1000.0); estimated number of hydrogen bonds that would be accepted by the solute (**donorHB =2.5** for both enantiomers; limits: 2.0–20.0); predicted octanol/water partition coefficient (**QPlogPo/w = 2.716** and **2.662** for (*R*)- and (*S*)-enantiomer, respectively; limits −2.0–6.5); predicted aqueous solubility. S, in mol/dm3, is the concentration of the solute’s saturated solution that is in equilibrium with crystalline solid (**QPlogS = −4.852** and **−5.125** for (*R*)- and (*S*)-enantiomer, respectively; limits −6.5–0.5); Van der Waals surface area of polar nitrogen and oxygen atoms (**PSA =107.282** and **107.713** for (*R*)- and (*S*)-enantiomer, respectively; limits 7.0–200.0); predicted brain/blood partition coefficient (**QPlog BB = −1.083** and **−1.289** for (*R*)- and (*S*)-enantiomer, respectively; limits −3.0–1.2); number of violations of Lipinski's Rule Of Five (molecular weight <500, QPlogPo/w < 5, number of hydrogen bond donor ≤5, number of hydrogen bond acceptors HB ≤10; **ROF =0** for both enantiomers); number of violations of Jorgensen's rule of three (QPlogS > −5.7, QPCaco >22 nm/s, number of primary metabolites <7; **ROT =1** for both enantiomers).

## Conclusions

We are persuaded that the MTSM approach is the most appropriate therapeutic strategy to design new drugs for AD therapy. In this context, Lipinski’s rules must be kept in mind during drug discovery to ensure molecular properties concerning pharmacokinetics will enable candidate drugs to have a greater opportunity of achieving the market. Based on this concept, new single dual-targeted chemical entities have been designed and synthesized by juxtaposition of molecular frameworks from KA and tacrine. The new KTs are small molecules showing ChE inhibitory activity, antioxidant, and neuroprotective properties. Particularly, **KT2d** [IC_50_ (hAChE) = 4.52 ± 0.24 µM; TE = 4.79], is six-fold less-hepatotoxic than tacrine at 1000 µM, nontoxic for SH-SY5Y cells at 10 µM, exhibiting significant neuroprotection against A*β*_1–40_ at 3 µM and 10 µM; thus, **KT2d** is a potential new hit agent that deserves further investigation for AD therapy. Work is now in progress in our laboratory to separate the enantiomers of the racemic mixture of compound **KT2d** in order to analyze their corresponding *in vitro* and *in vivo* biological properties. Results will be reported elsewhere.

## Supplementary Material

Supporting_Information_JEIMC_.pdf
