# Supplementary material for: Synthesis and biological assessment of KojoTacrines as new agents for Alzheimer’s disease therapy
Source: J Enzyme Inhib Med Chem. 2018 Nov 27;34(1):163–70. doi: 10.1080/14756366.2018.1538136 (PMC6263107; doi:10.1080/14756366.2018.1538136)
Supplement: Supporting_Information_JEIMC_.pdf [file IENZ_A_1538136_SM6412.pdf]

Supporting information

Synthesis and biological assessment of KojoTacrines as new agents for Alzheimer's disease therapy

Contents

1. Experimental section…………………………………………………………S2-S9

2. Inhibition of *Ee*AChE, hACHE, eqBuChE, (IC_{50}, μM) and ORAC-FL

values for KTs **2a-l**, tacrine and ferulic acid……………………………………………….S10

3. ^{1}H and ^{13}C NMR spectra of Kojotacrines…..………………………………………S11-S22

4. Binding mode and interaction of (*R*)**-KT2d** with hAChE………………………………S23

5. Binding mode and interaction of (*S*)**-KT2d** with hAChE……………………................S23

6. ADME of compounds **KT2a-l** ……………………………………………...S24-S26

7. References…………………………………………………………………………...S27

1. Experimental Section

Chemistry. Reactions were monitored with TLC using precoated silica gel aluminum plat from Merck. Infrared spectra were recorded on a Perkin-Elmer Spectrum covering field 400–4000 cm^{-1}. ^{1}H and ^{13}C NMR spectra were recorded in dimethylsulfoxide (DMSO-d_6) solution on a Bruker spectrometer (^{1}H NMR at 400 MHz, ^{13}C NMR at 100 MHz). Elemental analysis was performed on Thermofinnigan Flash EA 1112. The chemical shifts are expressed in parts per million (ppm) and the multiplicities are indicated by the following abbreviations: s, singlet; d, doublet; dd, double doublet; t, triplet; q, quadruplet; m, multiplet, and coupling constants are expressed in Hz.

General procedure for known compounds **1a-l** [1–3]. The synthesis of compounds **1a-l** was performed using the method described by Piao [24] with some modifications. A solution of arylidenemalononitrile (0.01 mol) in EtOH (10 mL) was treated with kojic acid (0.01 mol) and triethylamine (0.5 mL). The reaction mixture was heated at 80 °C for 5 min until precipitation. The solid formed was collected by filtration and recrystallized to give the corresponding 2-amino-6-(hydroxymethyl)-8-oxo-4-aryl-4,8-dihydropyrano[3,2-*b*]pyran-3-carbonitrile.

Compounds (**1a, f-h, k**) [4], (**1b**) [2], (**1d, e, i**) [3], and (**1j**) [5] had been previously described.

2-Amino-6-(hydroxymethyl)-4-(2-methoxyphenyl)-8-oxo-4,8-dihydropyrano[3,2-*b*]pyran-3-carbonitrile (**1c**): Yield 63 %; mp 220-218 °C; IR (KBr) ν 3392 and 3286 (NH_2), 3351 (OH), 2222 (CN) cm^{-1}; ^{1}H NMR (DMSO-d$_6$, 400 MHz) δ 3.77 (s, 3H), 4.24 (dd, 1H, ^{2}J = 16.4 Hz, ^{4}J = 6.2 Hz), 4.56 (s, 1H), 5.39 (s, 1H, OH), 6.33 (s, 1H), 6.54 (br s , 2H, NH_2), 6.61-6.80 (m, 4H), 7.16 (d, 1H, J = 7.5 Hz); ^{13}C NMR (DMSO-d$_6$, 100 MHz) δ 39.2 (CH), 54.4 (OCH_3), 55.2 (CH_2), 58.3 (C), 110.3 (CH), 119.6 (CN), 125.6 (CH), 129.0 (CH), 133.3 (CH), 137.1 (C), 138.2 (C), 142.6 (C), 149.6 (C), 157.8 (C), 158.3 (C), 169.0 (C), 196.2 (C).

2-Amino-4-(2,6-dichlorophenyl)-6-(hydroxymethyl)-8-oxo-4,8-dihydropyrano[3,2-*b*]pyran-3-carbonitrile (**1l**): Yield 77 %; mp 216-4 °C; IR (KBr) ν 3367 and 3350 (NH_2), 3449 (OH), 2222

(CN) cm^{-1}; ^{1}H NMR (DMSO-d$_6$, 300 MHz) δ 4.14 (dd, 1H, J = 5.6 Hz, J = 16.1 Hz), 4.22 (dd, 1H, J = 5.4 Hz, J = 16 Hz), 5.26 (s, 1H), 5.49 (s, 1H, OH), 6.38 (s, 1H), 6.85 (br s , 2H, NH_2), 7.24-7.34 (m, 3H), 7.40 (d, 1H, J = 7.5 Hz); ^{13}C-NMR (DMSO-d$_6$, 75 MHz) δ 40.1 (CH), 56.1 (CH_2), 59.8 (C), 112.0 (CH), 119.8 (CN), 131.9 (CH), 132.7 (CH), 136.6 (CH), 139.9 (C), 148.5 (C), 155.1 (C), 158.3 (C), 159.4 (C), 168.4 (C), 169.7 (C), 195.5 (C).

General procedure for the synthesis of KTs **2a-l**. To a solution of the corresponding aminonitrile **1a-l** (1.0 equiv) in distilled 1,4-dioxane, $AlCl_3$ (1.5 equiv) and cyclohexanone (1.5 equiv) were added. The reaction mixture was heated at 110 °C for 2 h and monitored by TLC (CH_2Cl_2/MeOH, 8/2, v/v). When the reaction was complete, the reaction mixture was diluted with a solution of CH_2Cl_2/water (1/1), and treated with an aqueous solution of sodium hydroxide (10%) until pH 11 to 12. The mixture was stirred for 20 min and then extracted with CH_2Cl_2, dried over anhydrous sodium sulfate, filtered and the solvent was evaporated. The resultant solid was washed with ether ethyl to give the corresponding KTs **2a-l**.

11-Amino-2-(hydroxymethyl)-12-phenyl-7,9,10,12-tetrahydropyrano[2',3':5,6]pyrano[2,3-*b*] quinolin-4(8*H*)-one (**2a**). Following the general method, the reaction of compound **1a** [4] (0.5 g, 1.6 mmol) in 1,4-dioxane (30 mL) with cyclohexanone (0.23 g, 2.4 mmol) and $AlCl_3$ (0.31 g, 2.4 mmol), after 2 h, gave product **2a** (0.51 g, 77 %): mp > 260 °C; IR (KBr) ν 3468 (OH), 3355 and 3250 (NH_2), 1719 (C=O) cm^{-1}; ^{1}H NMR (DMSO-d$_6$, 400 MHz) δ 1.71 (m, 4H), 2.21-2.33 (m, 2H), 2.51-2.59 (m, 2H), 4.23-4.25 (m, 2H), 5.37 (s, 1H), 5.67 (br s, 3H, OH, NH_2), 6.31 (s , 1H), 7.25-7.31 (m, 5H_{arom}); ^{13}C NMR (DMSO-d$_6$, 100 MHz) δ 22.47 (CH_2), 22.72 (CH_2), 23.42 (CH_2), 32.54 (CH_2), 39.27 (CH), 59.75 (CH_2), 96.80 (C), 111.72 (CH), 112.92 (C), 128.02 (CH), 128.29 (2CH), 129.20 (2CH), 137.81 (C), 141.14 (C), 150.12 (C), 152.17 (C), 153.70 (C), 154.35 (C), 168.12 (C), 171.04 (C). Anal. Calcd for $C_{22}H_{20}N_2O_4$: C, 70.20; H, 5.36; N, 7.44. Found: C, 70.67; H, 5.22; N, 7.38.

11-Amino-2-(hydroxymethyl)-12-(p-tolyl)-7,9,10,12-tetrahydropyrano[2',3':5,6]pyrano[2,3-*b*] quinolin-4(8*H*)-one (**2b**) : Following the general method, the reaction of compound **1b** [2] (0.6 g, 1.9 mmol) in 1,4-dioxane (30 mL) with cyclohexanone (0.28 g, 2.8 mmol) and $AlCl_3$ (0.32 g, 2.8 mmol), after 2 h, produced compound **2b** (0.37 g, 50 %): mp >260 °C; IR (KBr) ν 3460 (OH), 3350 and 3248 (NH_2), 1715 (C=O) cm^{-1}; ^1H NMR (DMSO-d_6, 400 MHz) δ 1.71 (m, 4H), 2.24-2.33 (m, 6H, CH_3, 3H cyclo), 2.51-2.59 (m, 2H), 4.22-4.26 (m, 2H), 5.31 (s, 1H), 5.67 (br s, 2H, NH_2), 6.30 (s , 1H), 7.12-7.18 (m, $4H_{arom}$); ^{13}C NMR (DMSO-d_6, 100 MHz) δ 21.07 (CH_3), 22.49 (CH_2), 22.74 (CH_2), 23.42 (CH_2), 32.54 (CH_2), 39.38 (CH), 59.76 (CH_2), 96.93 (C), 111.67 (CH), 112.88 (C), 128.20 (2CH), 129.75 (2CH), 137.28 (C), 137.71 (C), 138.23 (C), 150.2 (C), 152.23 (C), 152.12 (C), 153.62 (C), 154.29 (C), 168.10 (C), 171.06 (C). Anal. Calcd for $C_{23}H_{22}N_2O_4$: C, 70.75; H, 5.68; N, 7.18. Found: C, 71.01; H, 5.57; N, 7.28.

11-Amino-2-(hydroxymethyl)-12-(2-methoxyphenyl)-7,9,10,12-tetrahydropyrano[2',3':5,6] pyrano[2,3-*b*]quinolin-4(8*H*)-one (**2c**). Following the general method, the reaction of compound **1c** (0.6 g, 1.9 mmol) in 1,4-dioxane (30 mL) with cyclohexanone (0.28 g, 2.8 mmol) and $AlCl_3$ (0.32 g, 2.8 mmol), after 2 h, afforded product **2c** (0.46 g, 63 %): mp >260 °C; IR (KBr) ν 3470 (OH), 3330 and 3250 (NH_2), 1725 (C=O) cm^{-1}; ^1H NMR (DMSO-d_6, 400 MHz) δ 1.77 (m, 4H), 2.09-2.16 (m, 1H), 2.20-2.33 (m, 1H), 2.50-2.56 (m, 2H), 3.86 (s, 3H, OCH_3), 4.11-4.26 (m, 2H), 5.41 (br s, 2H, NH_2), 5.47 (s, 1H), 5.33-5.66 (t, 1H, OH), 6.32 (s , 1H), 6.91-6.95 (m, $1H_{arom}$), 7.07-7.15 (m, $2H_{arom}$), 7.24-7.28 (m, $1H_{arom}$) ; ^{13}C NMR (DMSO-d_6, 100 MHz) δ 22.46 (CH_2), 22.71 (CH_2), 23.32 (CH_2), 32.47 (CH_2), 33.81 (CH), 56.54 (OCH_3), 59.69 (CH_2), 97.43 (C), 111.64 (CH), 112.43 (CH),112.70 (C),122.10 (CH), 128.87 (C), 129.64 (CH),129.97 (CH), 138.73 (C), 149.52 (C), 151.80 (C), 153.37 (C), 154.10 (C), 156.45 (C), 168.17 (C), 171.06 (C). Anal. Calcd for $C_{23}H_{22}N_2O_5$: C, 67.97; H, 5.46; N, 6.89. Found: C, 68.21; H, 5.55 ; N, 6.81.

11-Amino-2-(hydroxymethyl)-12-(3-methoxyphenyl)-7,9,10,12-tetrahydropyrano[2',3':5,6] pyrano[2,3-*b*]quinolin-4(8*H*)-one (**2d**). Following the general method, the reaction of compound **1d** [3] (0.5 g, 1.5 mmol) in 1,4-dioxane (30 mL) with cyclohexanone (0.23 g, 2.25 mmol) and $AlCl_3$ (0.31 g, 2.25 mmol), after 2 h, generated product **2d** (0.36 g, 59%): mp >260 °C; IR (KBr) ν 3470 (OH), 3330 and 3250 (NH_2), 1725 (C=O) cm^{-1}; ^1H NMR (DMSO-d_6, 400 MHz) δ 1.72 (m, 4H), 2.18-2.22 (m, 1H), 2.33-2.37 (m, 1H), 2.55-2.66 (m, 2H), 3.72 (s, 3H, OCH_3), 4.20-4.28 (m, 2H), 5.32 (s, 1H), 5.69 (br s, 2H, NH_2), 6.32 (s , 1H), 6.77-6.4 (m, 4H_{arom}), 6.97 (s, 1H_{arom}), 7.21-7.25 (m, 1H_{arom}); ^{13}C NMR (DMSO-d_6, 100 MHz) δ 22.48 (CH_2), 22.72 (CH_2), 23.43 (CH_2), 32.55 (CH_2), 55.50 (OCH_3), 59.78 (CH_2), 96.74 (C), 111.75 (CH), 112.72 (CH), 112.88 (C), 114.96 (CH), 120.23 (CH), 130.40 (CH), 137.86 (C), 142.65 (C), 150.00 (C), 152.17 (C), 153.71 (C), 154.31 (C), 159.71 (C), 168.10 (C), 171.04 (C). Anal. Calcd for $C_{23}H_{22}N_2O_5$: C, 67.97; H, 5.46; N, 6.89. Found: C, 68.21; H, 5.38; N, 7.01.

11-Amino-2-(hydroxymethyl)-12-(4-methoxyphenyl)-7,9,10,12-tetrahydropyrano[2',3':5,6] pyrano[2,3-*b*]quinolin-4(8*H*)-one (**2e**). Following the general method, the reaction of compound **1e** [3] (0.8 g, 2 mmol) in 1,4-dioxane (30 mL) with cyclohexanone (0.37 g, 3 mmol) and $AlCl_3$ (0.43 g, 3 mmol), after 2 h, afforded product **2e** (0.51 g, 52%): mp >260 °C; IR (KBr) ν 3468 (OH), 3341 and 3216 (NH_2), 1714 (C=O) cm^{-1}; ^1H NMR (DMSO-d_6, 400 MHz) δ 1.72 (m, 4H), 2.17-2.22 (m, 1H), 2.33-2.37 (m, 1H), 2.51-2.59 (m, 2H), 3.70 (s, 3H, OCH_3), 4.18-4.31 (m, 2H), 5.29 (s, 1H), 5.64-5,69 (br s, 2H, NH_2, t, 1H, OH), 6.30 (s , 1H), 6.86-6.88 (m, 2H_{arom}), 7.20-7.23 (m, 2H_{arom}); ^{13}C NMR (DMSO-d_6, 100 MHz) δ 22.49 (CH_2), 22.74 (CH_2), 23.43 (CH_2), 35.54 (CH_2), 38.48 (CH), 55.52 (OCH_3), 59.76 (CH_2), 97.03(C), 111.65 (CH), 112.86 (C), 114.56 (2CH), 129.39 (2CH), 133.12 (C), 137.61 (C), 150.35 (C), 152.10 (C), 153.59 (C), 154.27 (C), 159.06 (C), 168.07 (C), 172.01 (C). Anal. Calcd for $C_{23}H_{22}N_2O_5$: C, 67.97; H, 5.46; N, 6.89. Found: C, 67.75; H, 5.52; N, 6.98.

11-Amino-12-(2-fluorophenyl)-2-(hydroxymethyl)-7,9,10,12-tetrahydropyrano[2',3':5,6] pyrano[2,3-*b*]quinolin-4(8*H*)-one (**2f**). Following the general method, the reaction of compound **1f** [4] (0.8 g, 2 mmol) in 1,4-dioxane (30 mL) with cyclohexanone (0.37 g, 3 mmol) and $AlCl_3$ (0.43 g, 3 mmol), after 2 h, accomplished product **2f** (0.61 g, 61%): mp >260 °C; IR (KBr) ν 3426 (OH), 3355 and 3250 (NH_2), 1711 (C=O) cm^{-1}; ^1H NMR (DMSO-d_6, 400 MHz) δ 1.71 (m, 4H), 2.17-2.21 (m, 1H), 2.33-2.37 (m, 1H), 2.52-2.60 (m, 2H), 4.16-4.29 (m, 2H), 5.59 (s, 1H), 5.68 (br s, 3H, OH, NH_2), 6.34 (s , 1H), 7.14-7.31 (m, $2H_{arom}$), 7.33-7.36 (m, $1H_{arom}$), 7.47-7.51 (m, $1H_{arom}$); ^{13}C NMR (DMSO-d_6, 100 MHz) δ 21.86 (CH_2), 22.05 (CH_2), 22.84 (CH_2), 34.53 (CH_2), 38.89 (CH), 59.25 (CH_2), 95.29 (C), 111.32 (CH), 112.67 (C), 116.04 (CH), 124.82 (CH), 126.60 (C), 130.12 (C), 130.78 (C), 137.76 (C), 147.72 (C), 152.07 (C), 152.70 (C), 153.44 (C), 160.43 (C-F, J = 246Hz), 167.86 (C), 170.43 (C). Anal. Calcd for $C_{22}H_{19}FN_2O_4$: C, 67.00; H, 4.86; N, 7.10. Found: C, 67.25; H, 4.93; N, 7.22.

11-Amino-12-(3-fluorophenyl)-2-(hydroxymethyl)-7,9,10,12-tetrahydropyrano[2',3':5,6] pyrano[2,3-*b*]quinolin-4(8*H*)-one (**2g**). Following the general method, the reaction of compound **1g** [4] (0.5 g, 1.5 mmol) in 1,4-dioxane (30 mL) with cyclohexanone (0.23 g, 2.25 mmol) and $AlCl_3$ (0.31 g, 2.25 mmol), after 2 h, rendered compound **2g** (0.35 g, 56%): mp >260 °C; IR (KBr) ν 3455 (OH), 3336 and 3257 (NH_2), 1709 (C=O) cm^{-1}; ^1H NMR (DMSO-d_6, 400 MHz) δ 1.72 (m, 4H), 2.19-2.23 (m, 1H), 2.33-2.38 (m, 1H), 2.51-2.60 (m, 2H), 4.20-4.30 (m, 2H), 5.41 (s, 1H), 5.66-5.69 (t, 1H, OH), 5.76 (br s, 2H, NH_2), 6.33 (s , 1H), 7.05-7.20 (m, $2H_{arom}$), 7.22-7.23 (m, $1H_{arom}$), 7.34-7.38 (m, $1H_{arom}$); ^{13}C NMR (DMSO-d_6, 100 MHz) δ 22.44 (CH_2), 22.68 (CH_2), 23.42 (CH_2), 32.51 (CH_2), 39.98 (CH), 59.76 (CH_2), 96.32 (C), 111.79 (CH), 113.0 (C), 114.83 (CH), 115.50 (CH), 124.26 (CH), 131.34 (CH), 137.92 (C), 143.82 (C), 149.40 (C), 152.26 (C), 153.89 (C), 154.21 (C), 162.49 (C-F, J=243), 168.19 (C), 171.01 (C). Anal. Calcd for $C_{22}H_{19}FN_2O_4$: C, 67.00; H, 4.86; N, 7.10. Found: C, 67.26; H, 4.79; N, 6.99.

11-Amino-12-(4-fluorophenyl)-2-(hydroxymethyl)-7,9,10,12-tetrahydropyrano[2',3':5,6] pyrano[2,3-*b*]quinolin-4(8*H*)-one (**2h**). Following the general method, the reaction of compound **1h** [4] (0.5 g, 1.5 mmol) in 1,4-dioxane (30 mL) with cyclohexanone (0.23 g, 2.25 mmol) and $AlCl_3$ (0.31 g, 2.25 mmol), after 2 h, afforded product **2h** (0.40 g, 64%): mp >260 °C; IR (KBr) ν 3471(OH), 3342 and 3217 (NH_2), 1720 (C=O) cm^{-1}; ^1H NMR (DMSO-d_6, 400 MHz) δ 1.72 (m, 4H), 2.18-2.22 (m, 1H), 2.33-2.38 (m, 1H), 2.51-2.58 (m, 2H), 4.18-4.25 (m, 2H), 5.39 (s, 1H), 5.66-5.76 (br s, 2H, NH_2, t, 1H, OH), 7.1-7.17 (m , $2H_{arom}$), 7.32-7.35 (m, $2H_{arom}$); ^{13}C NMR (DMSO-d_6, 100 MHz) δ 22.46 (CH_2), 22.71 (CH_2), 23.42 (CH_2), 32.55 (CH_2), 38.51 (CH), 59.75 (CH_2), 96.62 (C), 111.71 (CH), 112.96 (C), 115.87 (2CH), 130.22 (2CH), 137.27 (C), 137.75 (C), 149.81 (C), 152.17 (C), 153.82 (C), 154.27 (C), 161.91 (C-F, J =242Hz), 168.14 (C), 171.03 (C). Anal. Calcd for $C_{22}H_{19}FN_2O_4$: C, 67.00; H, 4.86; N, 7.10. Found: C, 66.81; H, 4.81; N, 7.19.

11-Amino-12-(2-chlorophenyl)-2-(hydroxymethyl)-7,9,10,12-tetrahydropyrano[2',3':5,6] pyrano[2,3-*b*]quinolin-4(8*H*)-one (**2i**). Following the general method, the reaction of compound **1i** [3] (0.5 g, 1.5 mmol) in 1,4-dioxane (30 mL) with cyclohexanone (0.23 g, 2.25 mmol) and $AlCl_3$ (0.31 g, 2.25 mmol), after 2 h, gave product **2i** (0.41 g, 70%): mp >260 °C; IR (KBr) ν 3471(OH), 3351 and 3260 (NH_2), 1719 (C=O) cm^{-1}; ^1H NMR (DMSO-d_6, 400 MHz) δ 1.71 (m, 4H), 2.17-2.21 (m, 1H), 2.231-2.35 (m, 1H), 2.51-2.68 (m, 2H), 4.12-4.25 (m, 2H), 5.22 (br s, 2H, NH_2), 5.65 (s, 1H), 6.32 (s , 1H), 7.30-7.39 (m, $1H_{arom}$), 7.46-7.48 (m, $1H_{arom}$); ^{13}C NMR (DMSO-d_6, 100 MHz) δ 22.39 (CH_2), 22.66 (CH_2), 23.33 (CH_2), 32.50 (CH_2), 38.08 (CH), 59.68 (CH_2), 96.38 (C), 111.66 (CH), 113.24 (C), 128.68 (CH), 130.23 (CH), 130.53 (CH), 131.74 (C), 132.76 (C), 137.75 (C), 138.23 (C), 148.04 (C), 151.96 (C), 154.10 (C), 154.16 (C), 168.30 (C), 170.94 (C). Anal. Calcd for $C_{22}H_{19}ClN_2O_4$: C, 64.32; H, 4.66; N, 6.82. Found: C, 64.45; H, 4.80; N, 6.77.

11-Amino-12-(3-chlorophenyl)-2-(hydroxymethyl)-7,9,10,12-tetrahydropyrano[2',3':5,6] pyrano[2,3-*b*]quinolin-4(8*H*)-one **(2j).** Following the general method, the reaction of compound **1j** [5] (0.5 g, 1.5 mmol) in 1,4-dioxane (30 mL) with cyclohexanone (0.23 g, 2.25 mmol) and $AlCl_3$ (0.31 g, 2.25 mmol), after 2 h, yielded product **2j** (0.45 g, 74%): mp >260 °C; IR (KBr) ν 3432(OH), 3352 and 3219 (NH_2), 1717 (C=O) cm^{-1}; ^1H NMR (DMSO-d_6, 400 MHz) δ 1.72 (m, 4H), 2.22-2.33 (m, 2H), 2.59 (m, 2H), 4.26 (m, 2H), 5.40 (s, 1H), 5.68 and 577 (br, s, 1H, OH and br s, 2H, NH_2), 6.33 (s , 1H), 7.21-7.45 (m, $4H_{arom}$); ^{13}C NMR (DMSO-d_6, 100 MHz) δ 22.43 (CH_2), 22.68 (CH_2), 23.42 (CH_2), 32.55 (CH_2), 39.36 (CH), 59.77 (CH_2), 96.31 (C), 111.82 (CH), 113.03 (C), 126.95 (CH), 128.22 (CH), 131.26 (CH), 133.59 (CH), 137.98 (C), 143.52 (C), 149.35 (C), 152.19 (C), 153.99 (C), 154.20 (C), 168.20 (C), 171.02 (C). Anal. Calcd for $C_{22}H_{19}ClN_2O_4$: C, 64.32; H, 4.66; N, 6.82. Found: C, 64.49; H, 4 .72; N, 6.91.

11-Amino-12-(3-bromophenyl)-2-(hydroxymethyl)-7,9,10,12-tetrahydropyrano[2',3':5,6] pyrano[2,3-*b*]quinolin-4(8*H*)-one (**2k**). Following the general method, the reaction of compound **1k** [4] (0.5 g, 1.5 mmol) in 1,4-dioxane (30 mL) with cyclohexanone (0.23 g, 2.25 mmol) and $AlCl_3$ (0.31 g, 2.25 mmol), after 2 h, afforded product **2k** (0.41 g, 69%): mp >260 °C; IR (KBr) ν 3444 (OH), 3359 and 3243 (NH_2), 1719 (C=O) cm^{-1}; ^1H NMR (DMSO-d_6, 400 MHz) δ 1.72 (m, 4H), 2.18-2.23 (m, 1H), 2.33-2.38 (m, 1H), 2.52-2.68 (m, 2H), 4.20-4.32 (m, 2H), 5.38 (s, 1H), 5.66-5.68 (t, 1H, OH), 5.77 (br s, 2H, NH_2), 6.33 (s , 1H), 7.23-7.31 (m, 2H, $2H_{arom}$), 7.45-7.47 (m, 1H, $1H_{arom}$), 7.59-7.60 (m, 1H, $1H_{arom}$); ^{13}C NMR (DMSO-d_6, 100 MHz) δ 21.96 (CH_2), 22.21 (CH_2), 22.96 (CH_2), 32.08 (CH_2), 38.47 (CH), 59.30 (CH_2), 95.86 (C), 111.36 (CH), 112.57 (C), 121.80 (C), 126.86 (CH), 130.55 (CH), 130.58 (CH), 131.10 (CH), 137.52 (C), 143.31 (C), 148.91 (C), 151.71 (C), 153.53 (C), 153.72 (C), 167.74 (C), 170.55 (C). Anal. Calcd for $C_{22}H_{19}BrN_2O_4$: C, 58.04; H, 4.21; N, 6.15. Found: C, 58.30; H, 4.28; N, 6.19.

11-Amino-12-(2,6-dichlorophenyl)-2-(hydroxymethyl)-7,9,10,12-tetrahydropyrano [2',3':5,6] pyrano[2,3-*b*]quinolin-4(8*H*)-one (**2l**). Following the general method, the reaction of compound

1l (0.8 g, 2 mmol) in 1,4-dioxane (30 mL) with cyclohexanone (0.37 g, 3 mmol) and $AlCl_3$ (0.43 g, 3 mmol), after 2 h, provided compound **2l** (0.74 g, 77%): mp >260 °C; IR (KBr) ν 3470 (OH), 3347 and 3235 (NH_2), 1717 (C=O) cm^{-1}; ^1H NMR (DMSO-d_6, 400 MHz) δ 1.71 (m, 4H), 2.22-2.29 (m, 2H), 2.51-2.58 (m, 2H), 4.13-4.18 (m, 2H), 4.94 (br s, 2H, NH_2), 5.63 (br s, 1H, OH), 6.21 (s , 1H), 6.33 (s , 1H), 7.43 (m, $2H_{arom}$), 7.67 (m, $1H_{arom}$); ^{13}C NMR (DMSO-d_6, 100 MHz) δ 21.92 (CH_2), 22.18 (CH_2), 22.77 (CH_2), 37.16 (CH_2), 38.89 (CH), 59.19 (CH_2), 94.72 (C), 111.23 (CH), 112.82 (C), 128.99 (2CH), 130.92 (CH), 131.33 (CH), 132.42 (2C), 134.98 (C), 135.22 (C), 138.84 (C), 144.83 (C),151.27 (C), 153.53 (C), 154.12 (C), 167.76 (C), 170.22 (C). Anal. Calcd for $C_{22}H_{18}Cl_2N_2O_4$: C, 59.34; H, 4.07; N, 6.29. Found: C, 59.61; H, 4 .01; N, 6.39.

2. Inhibition of *Ee*AChE, hACHE, eqBuChE, (IC_{50}, μM) and ORAC-FL values for KTs **2a-l**, tacrine and ferulic acid

Table 1S. Inhibition of *Ee*AChE, hACHE, eqBuChE, (IC_{50}, μM) and ORAC-FL values for KTs **2a-l**, tacrine and ferulic acid (FA).

**KT**	R	*Ee*AChE[a] (IC_{50}, μM)	*eq*BuChE (IC_{50}, μM)	hAChE (IC_{50} μM)	ORAC
**2a**	H	3.30±0.09	12.15±0.09	-[b]	2.58±0.22
**2b**	4-CH_3	1.40±0.00	10.09±0.09	13.7±1.7	2.96±0.18
**2c**	2-OCH_3	2.39±0.02	-[c]	-[c]	6.05±0.41
**2d**	**3-OCH_3**	**0.64±0.06**	**-[c]**	4.52±0.24	**4.79±0.39**
**2e**	4-OCH_3	1.14±0.02	7.57±0.13	-[d]	4.46±0.27
**2f**	2-F	2.11±0.01	6.69±0.10	-[c]	3.85±0.42
**2g**	3-F	2.14±0.12	4.54±0.20	-[d]	4.34±0.17
**2h**	4-F	3.44±0.04	-[c]	-[d]	4.69±0.18
**2i**	2-Cl	0.76±0.09	8.54±0.12	-[d]	4.84±0.06
**2j**	3-Cl	0.82±0.05	4.19±0.11	-[d]	4.67±0.31
**2k**	3-Br	0.74±0.08	3.90±0.09	-[d]	2.61±0.11
**2l**	2,6-diCl	2.12±0.09	-[c]	-[d]	6.14±0.40
Tacrine	-	0.031±0.006	0.005±0.001	0.13±0.00	0.2±0.1
FA	-	-[d]	-[d]	-[d]	3.74±0.22

[a]Inhibition curves were obtained by nonlinear regression. *Ee*: electric eel, eq: equine. Each IC_{50} value is the mean ± SEM of quadruplicate of at least three different experiments. Results are expressed in μM; [c] % inhibition under 50% at 10μM. [d]Not determined.

3. ^{1}H and ^{13}C NMR spectra of Kojotacrines

^{1}H NMR spectra of KT2a

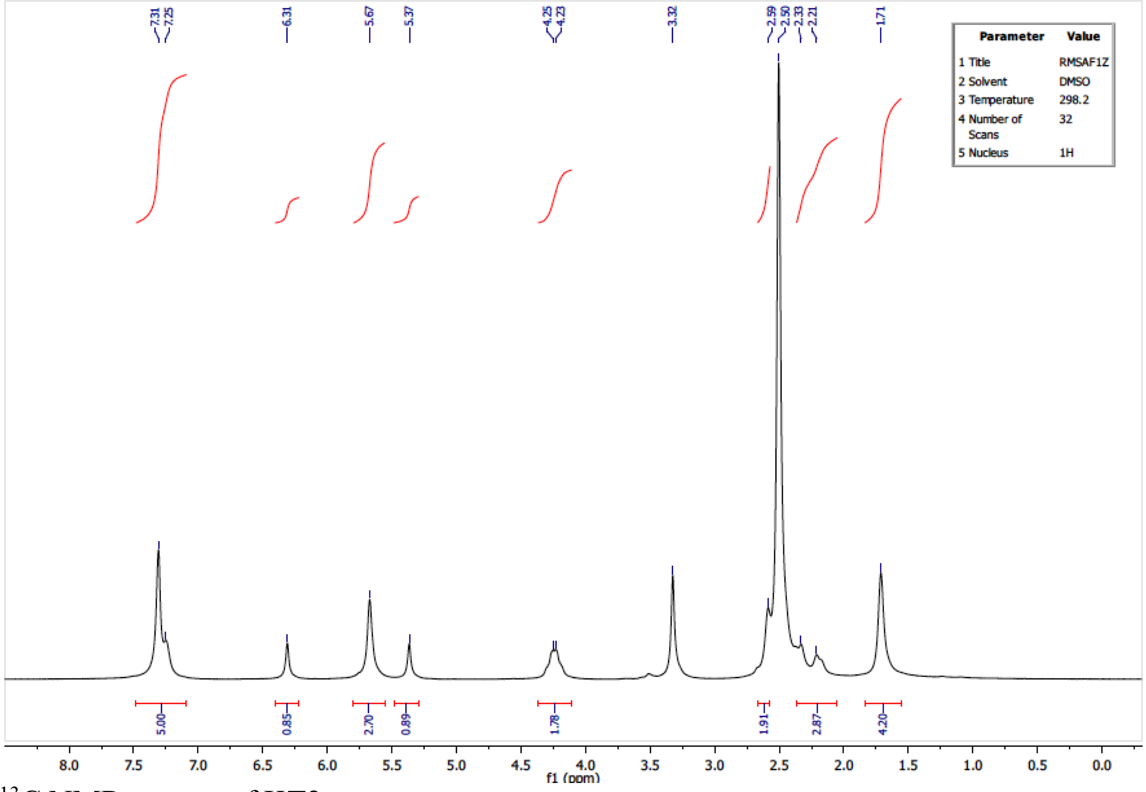

^{13}C NMR spectra of KT2a

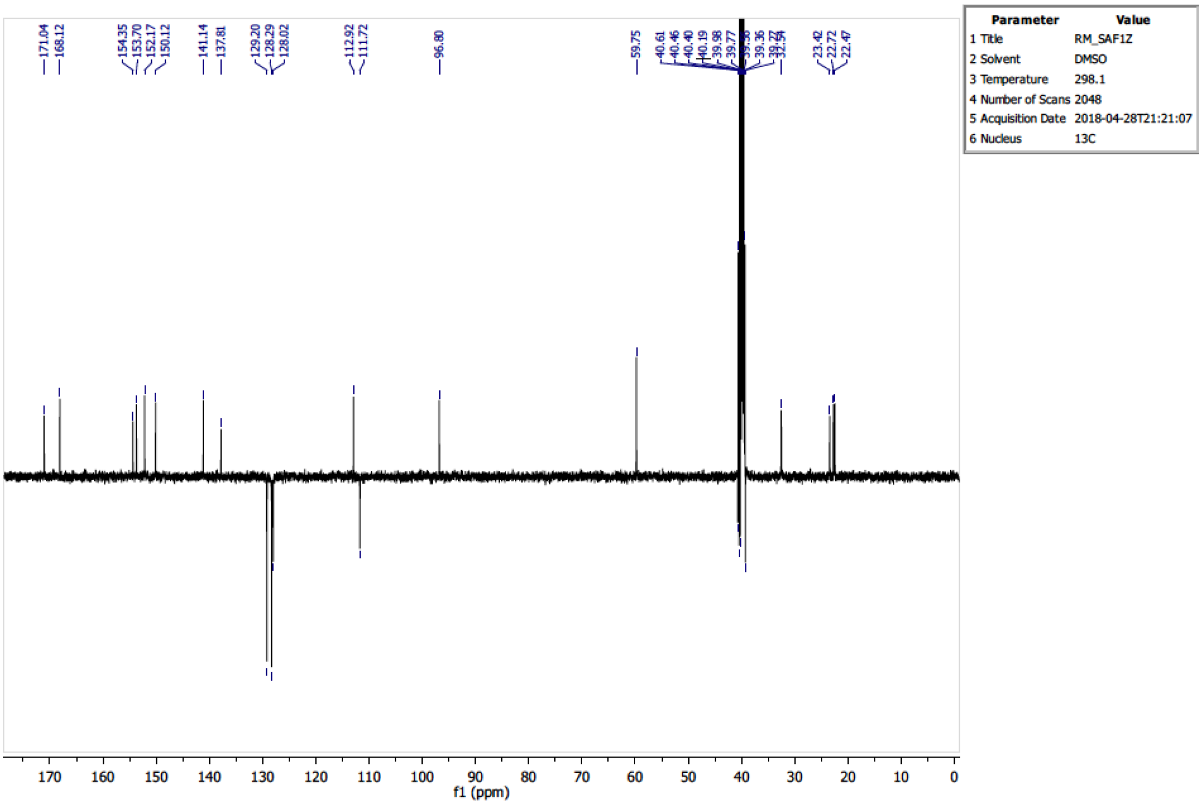

^{1}H NMR spectra of KT2b

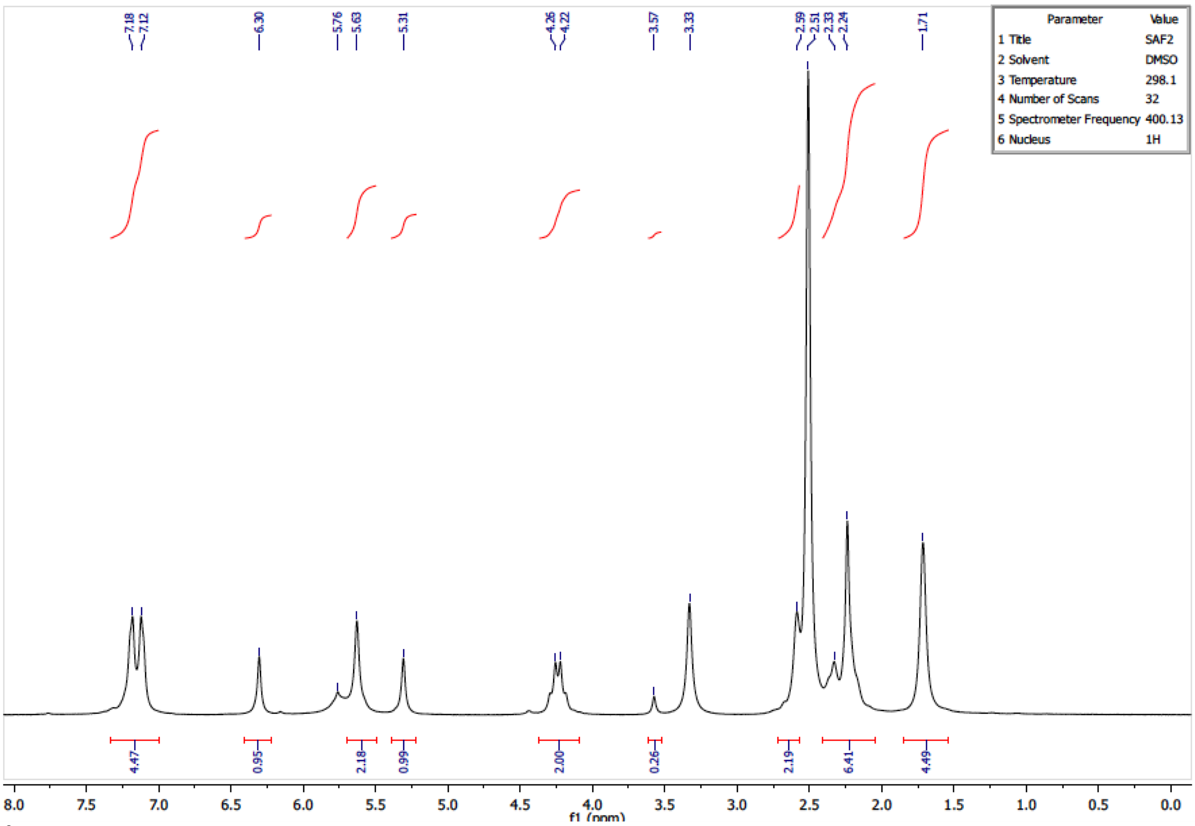

^{13}C NMR spectra of KT2b

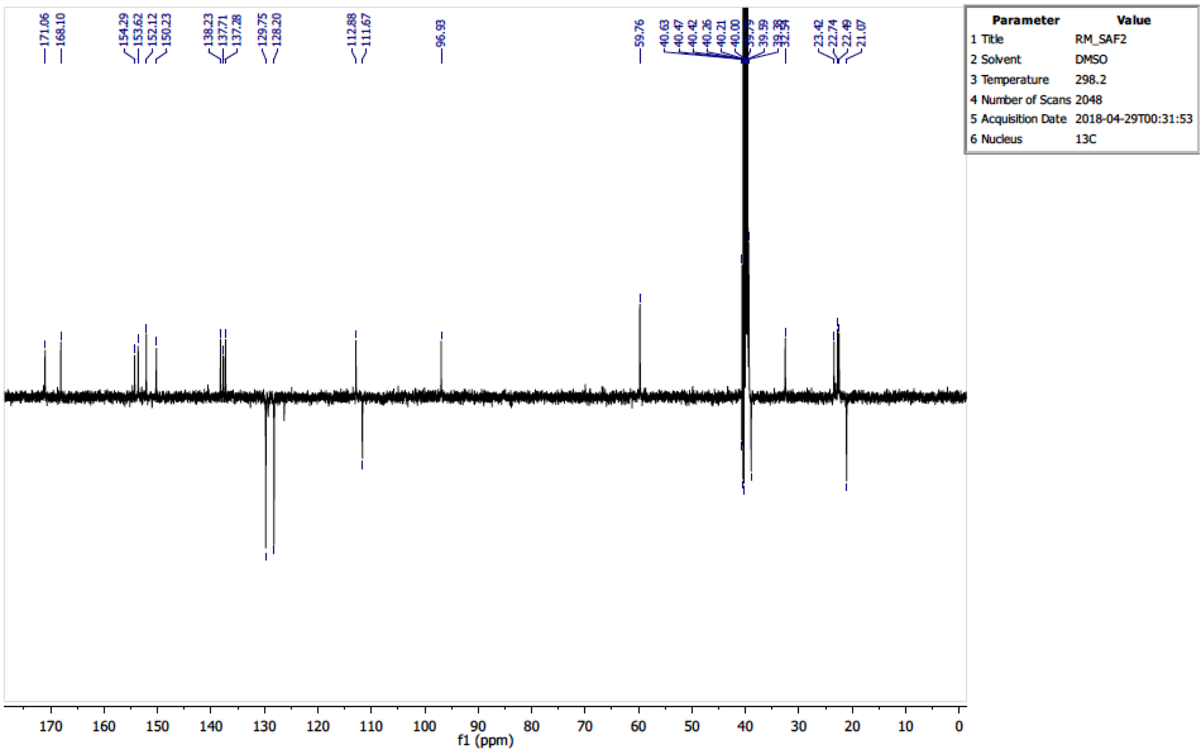

^{1}H NMR spectra of KT2c

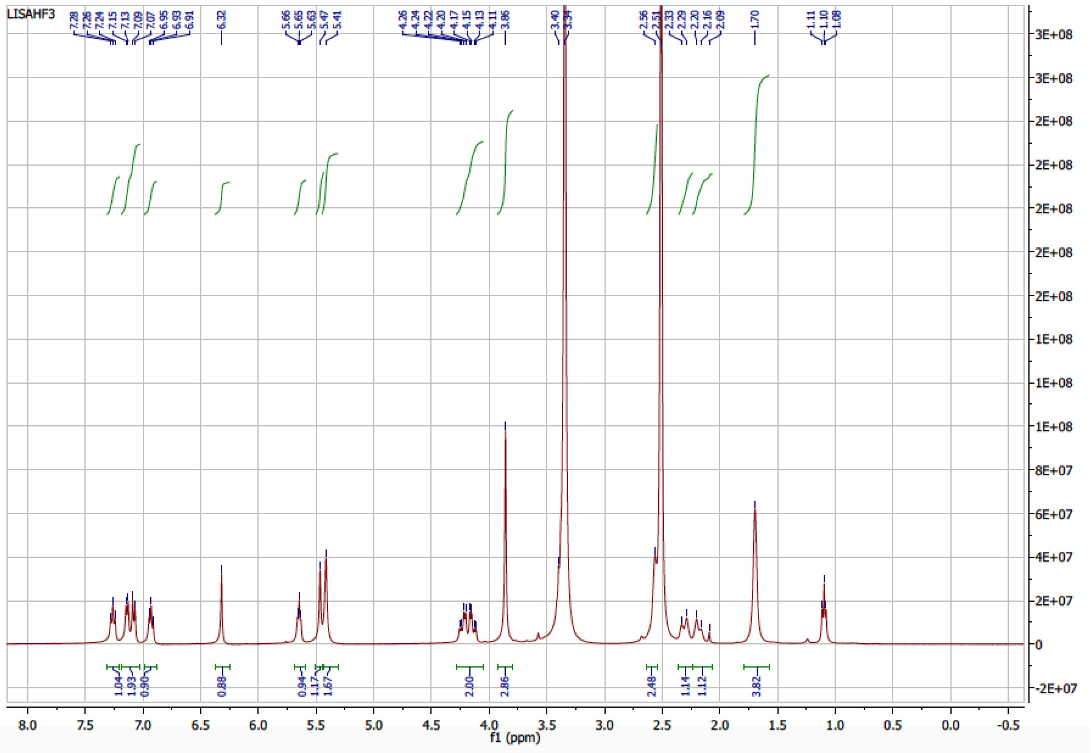

^{13}C NMR spectra of KT2c

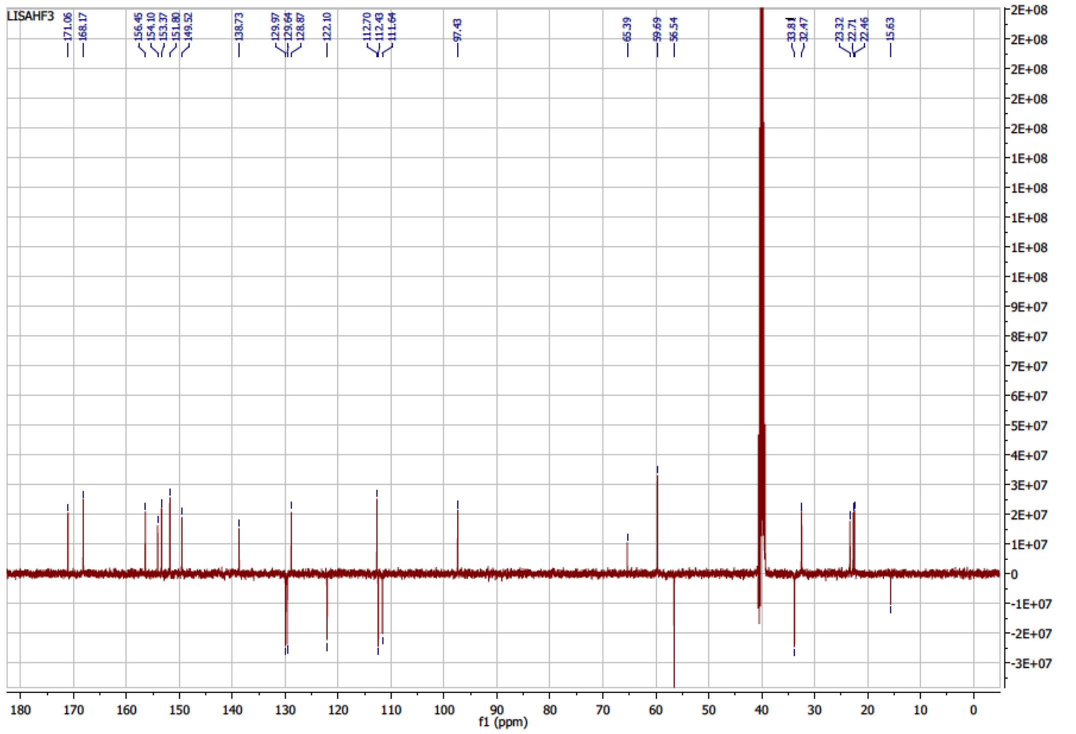

^{1}H NMR spectra of KT2d

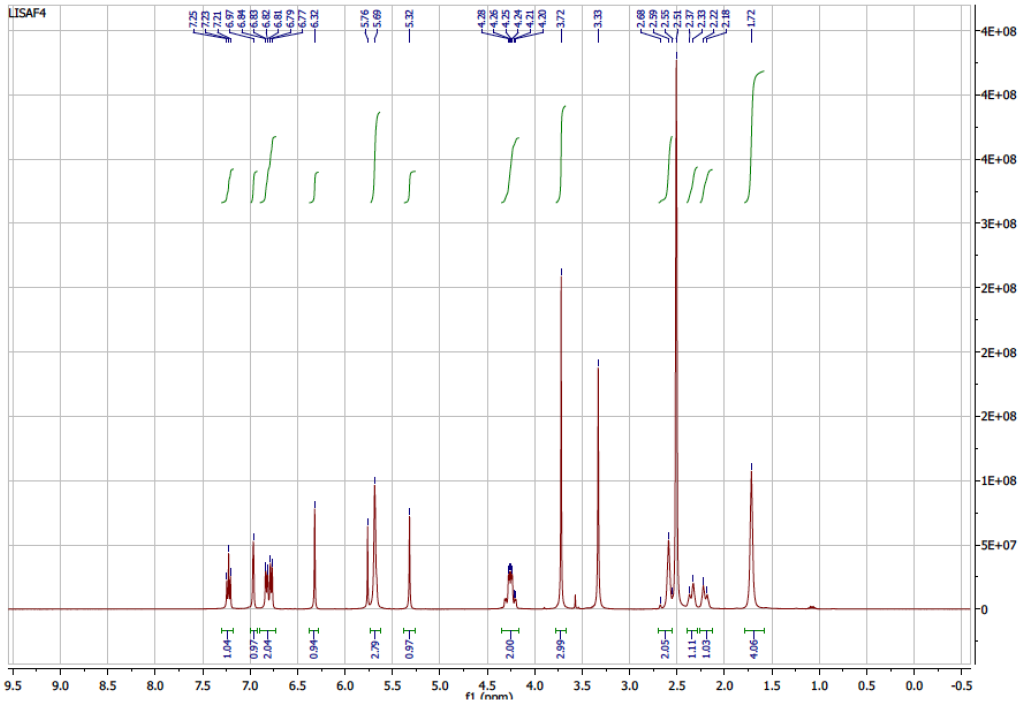

^{13}C NMR spectra of KT2d

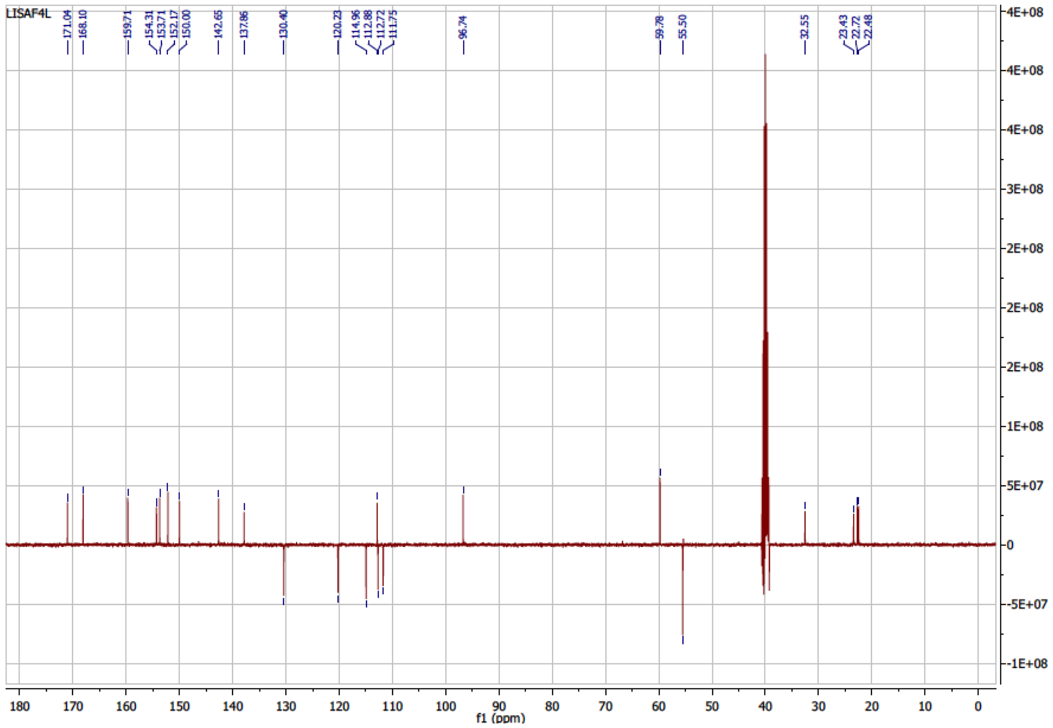

^{1}H NMR spectra of KT2e

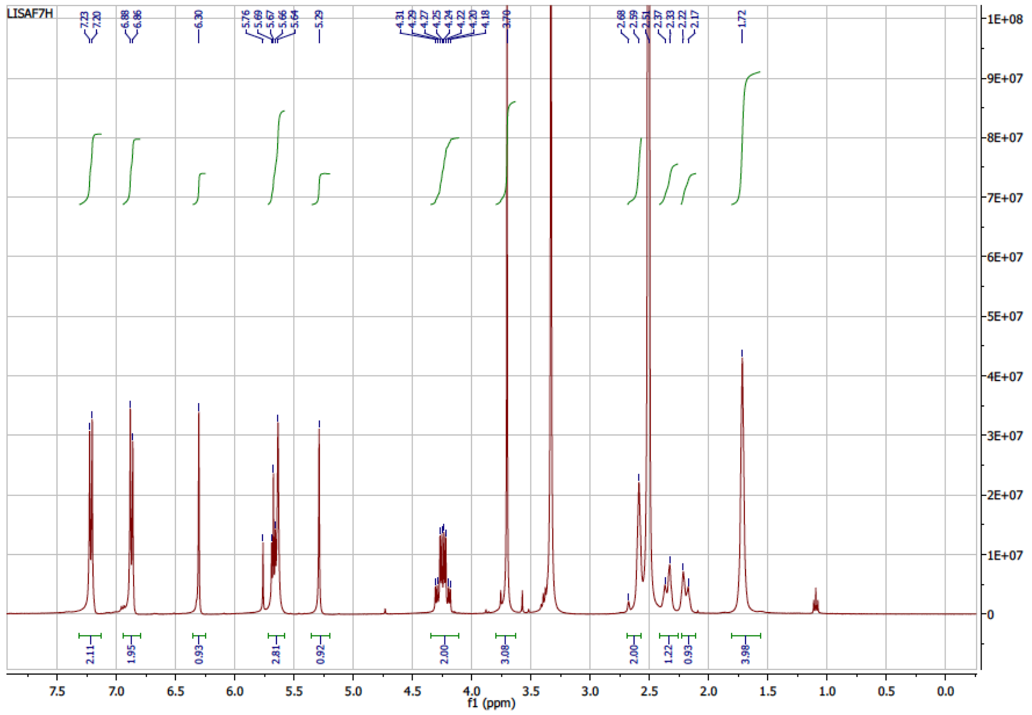

^{13}C NMR spectra of KT2e

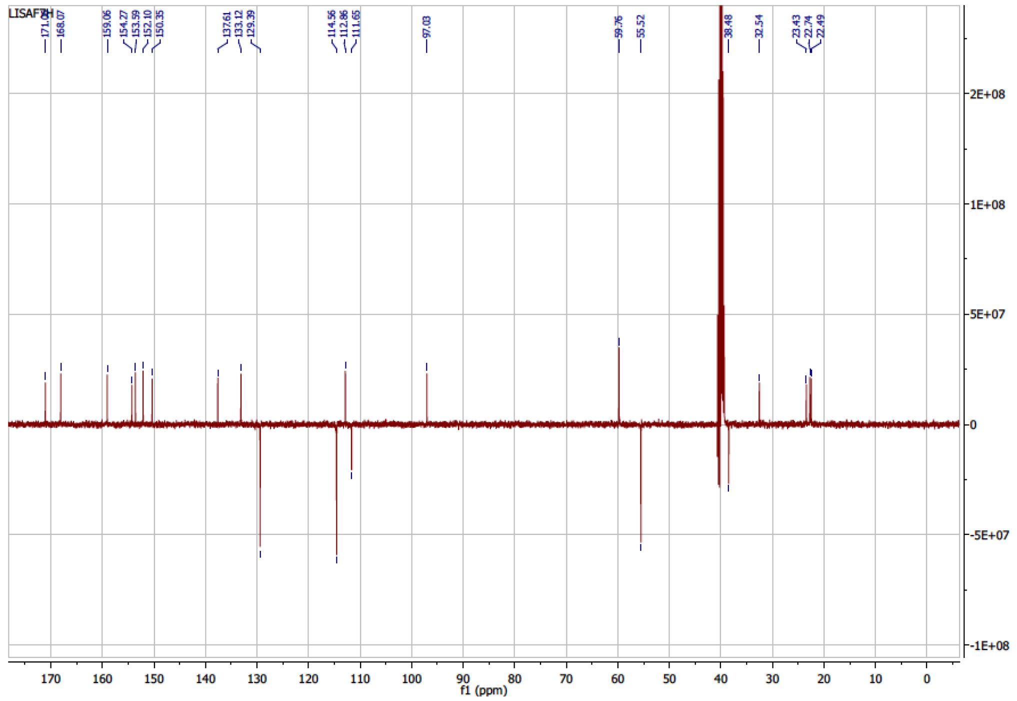

^{1}H NMR spectra of KT2f

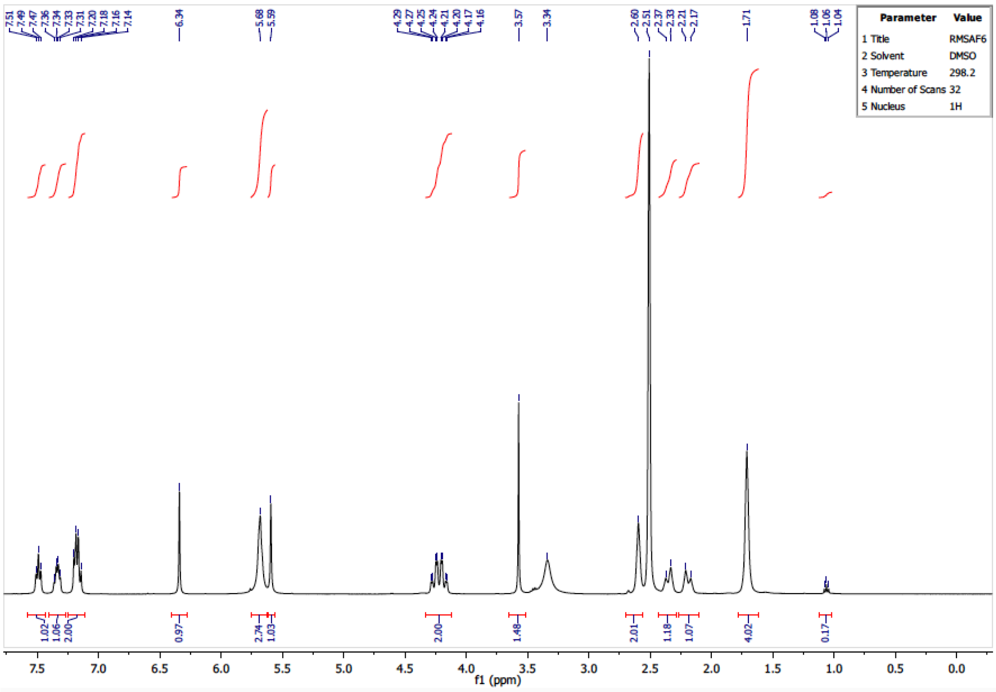

^{1}H NMR spectra of KT2f

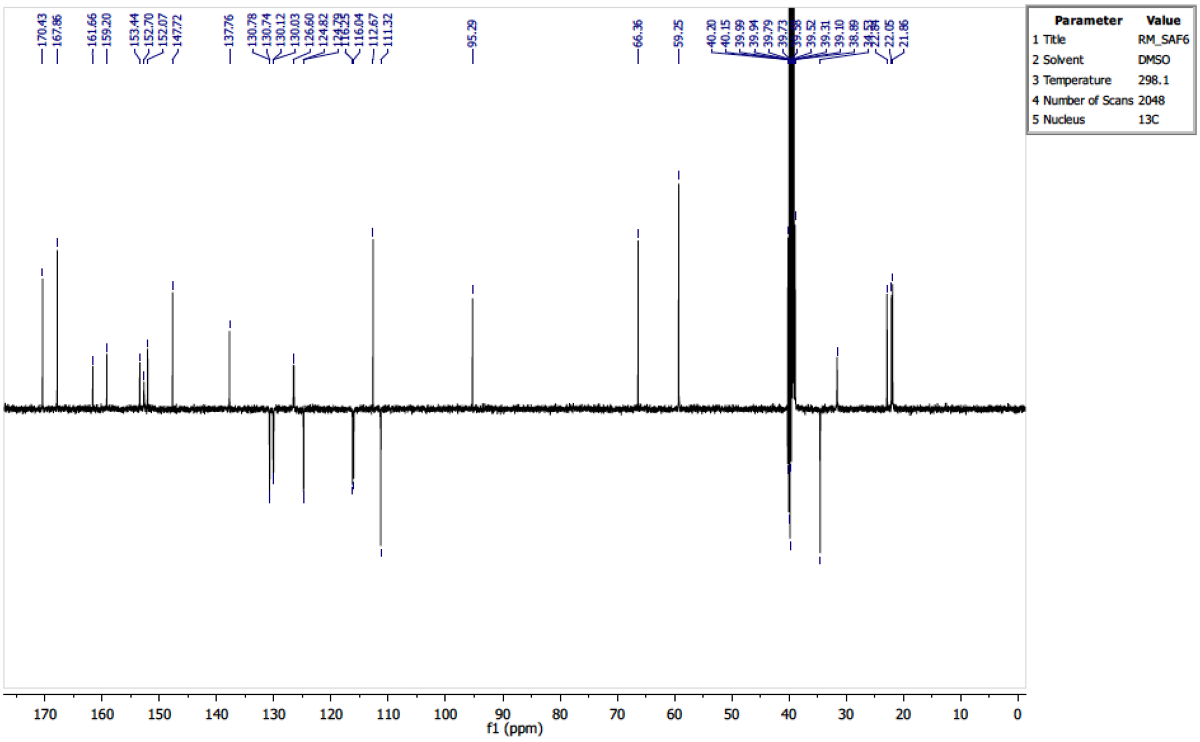

^{1}H NMR spectra of KT2g

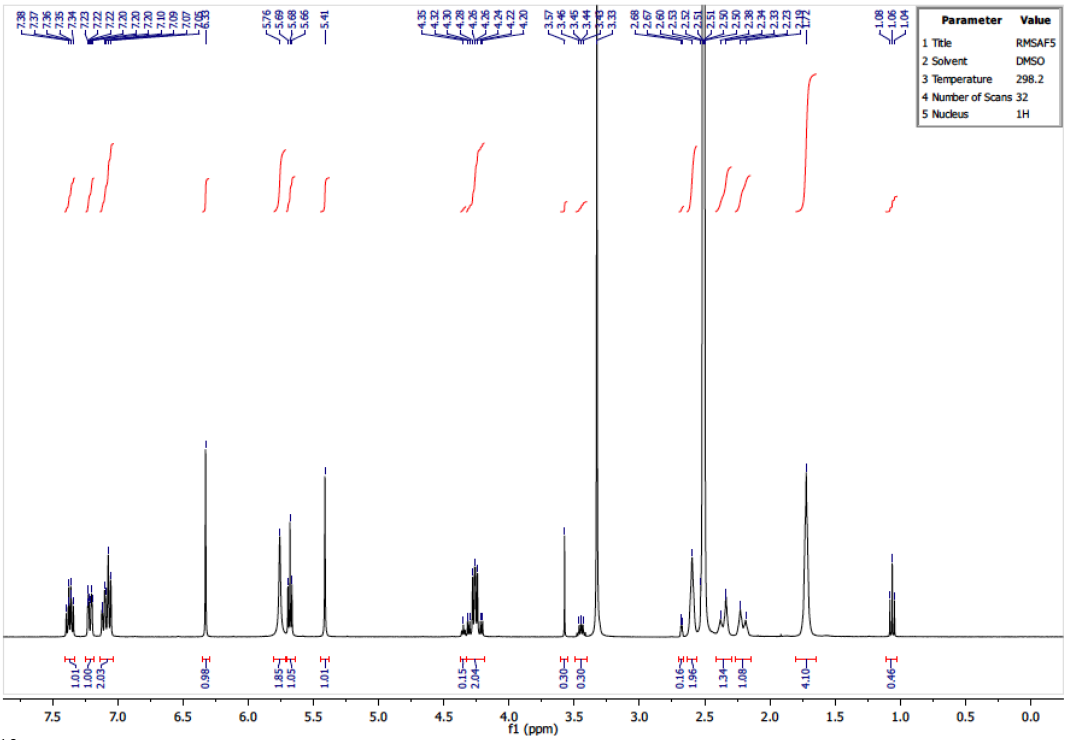

^{13}C NMR spectra of KT2g

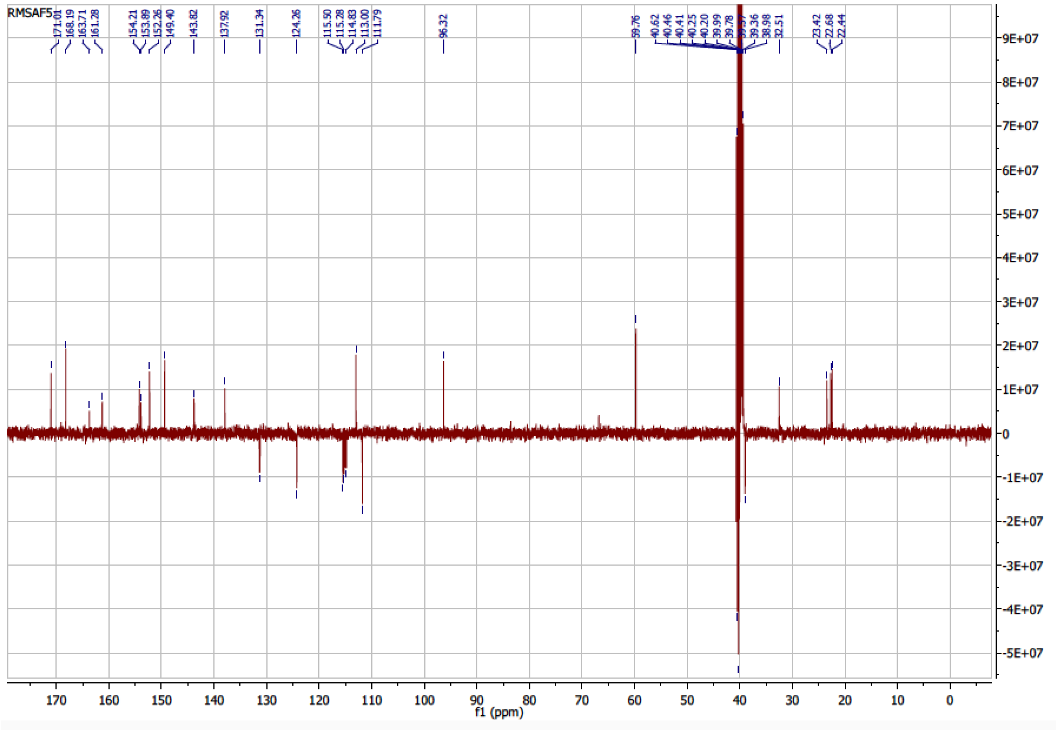

^{1}H NMR spectra of KT2h

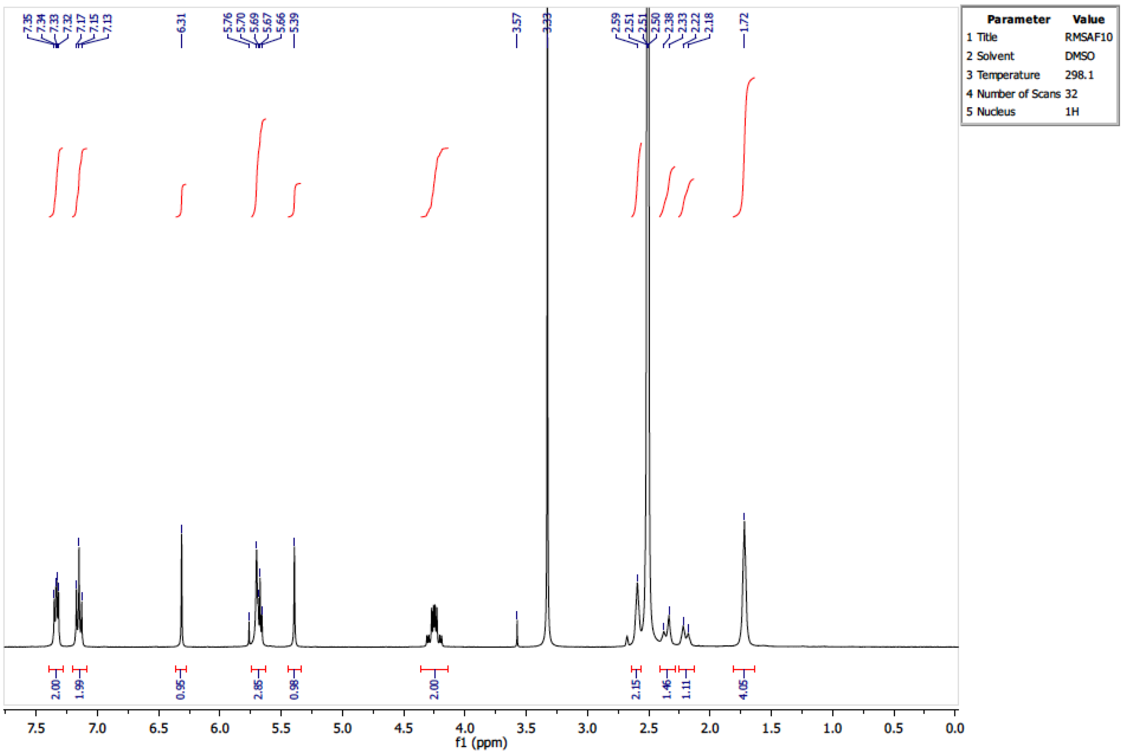

^{13}C NMR spectra of KT2h

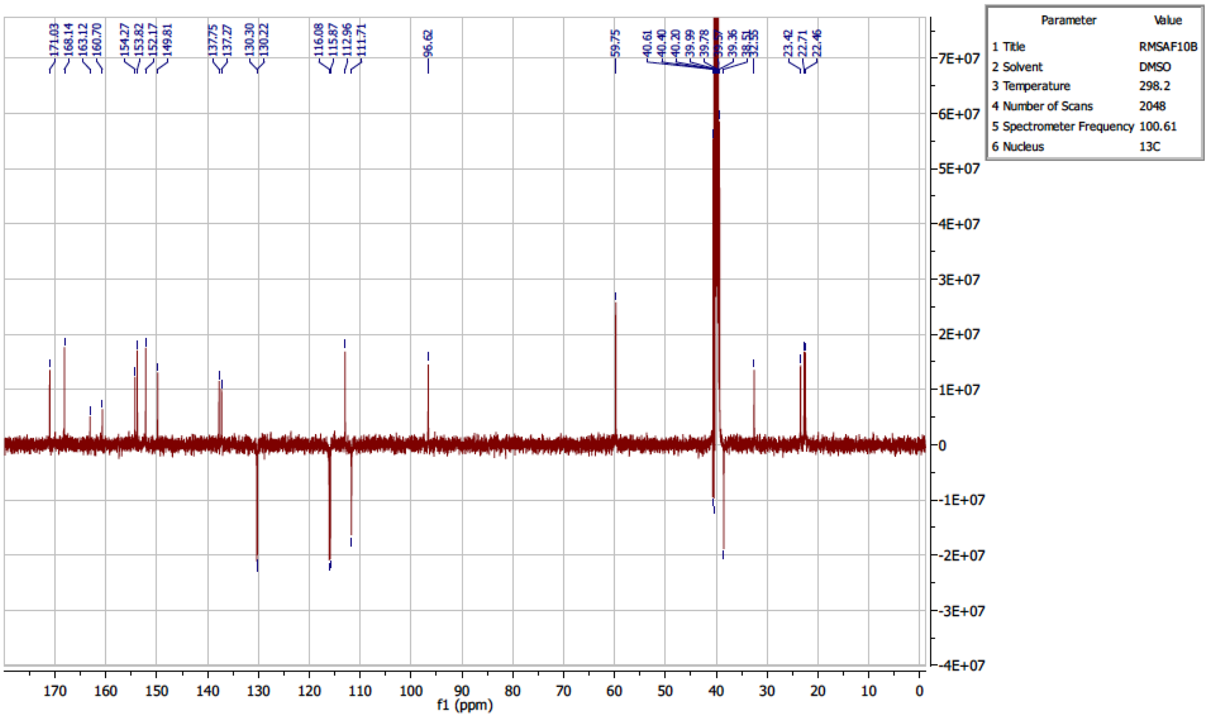

^{1}H NMR spectra of KT2i

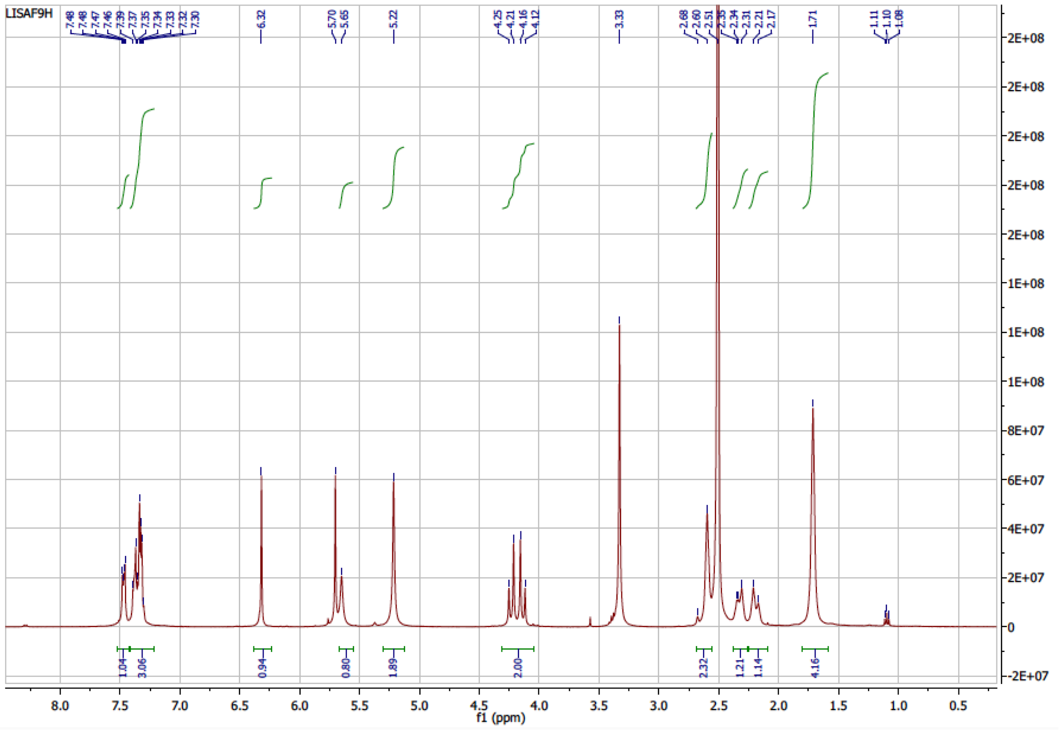

^{13}C NMR spectra of KT2i

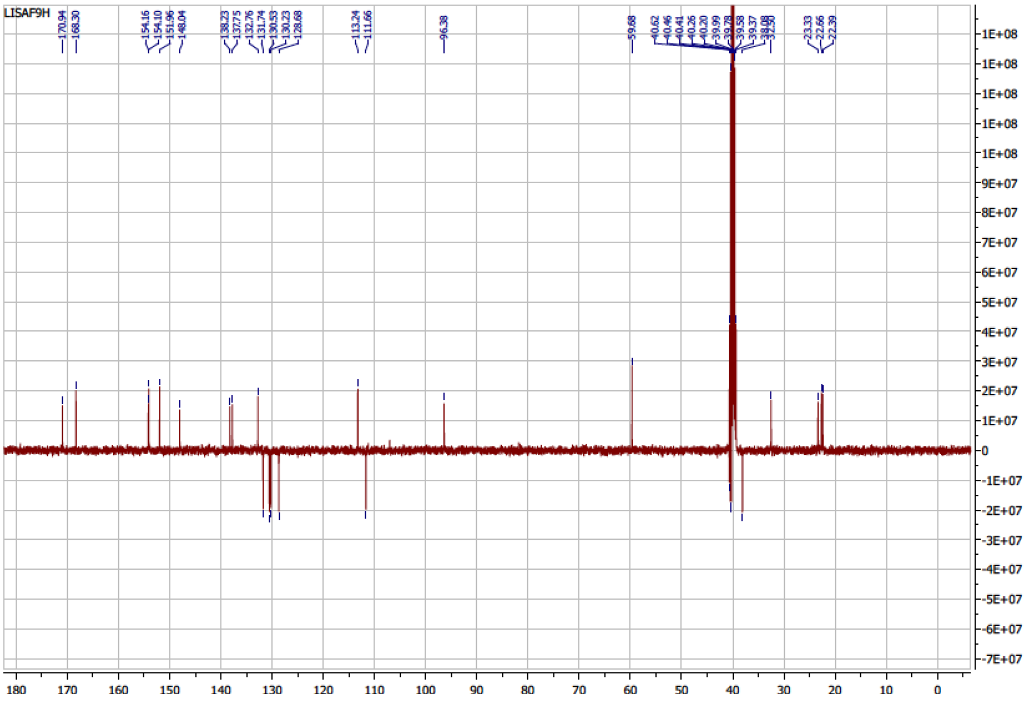

^{1}H NMR spectra of KT2j

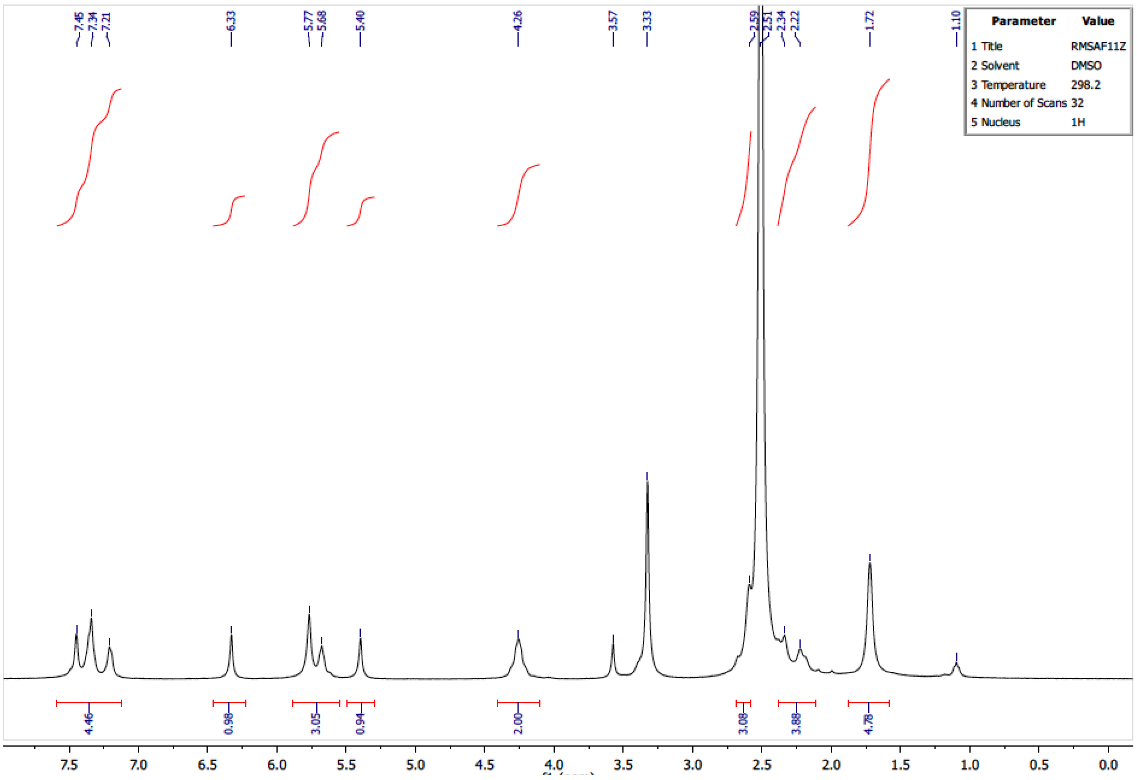

^{13}C NMR spectra of KT2j

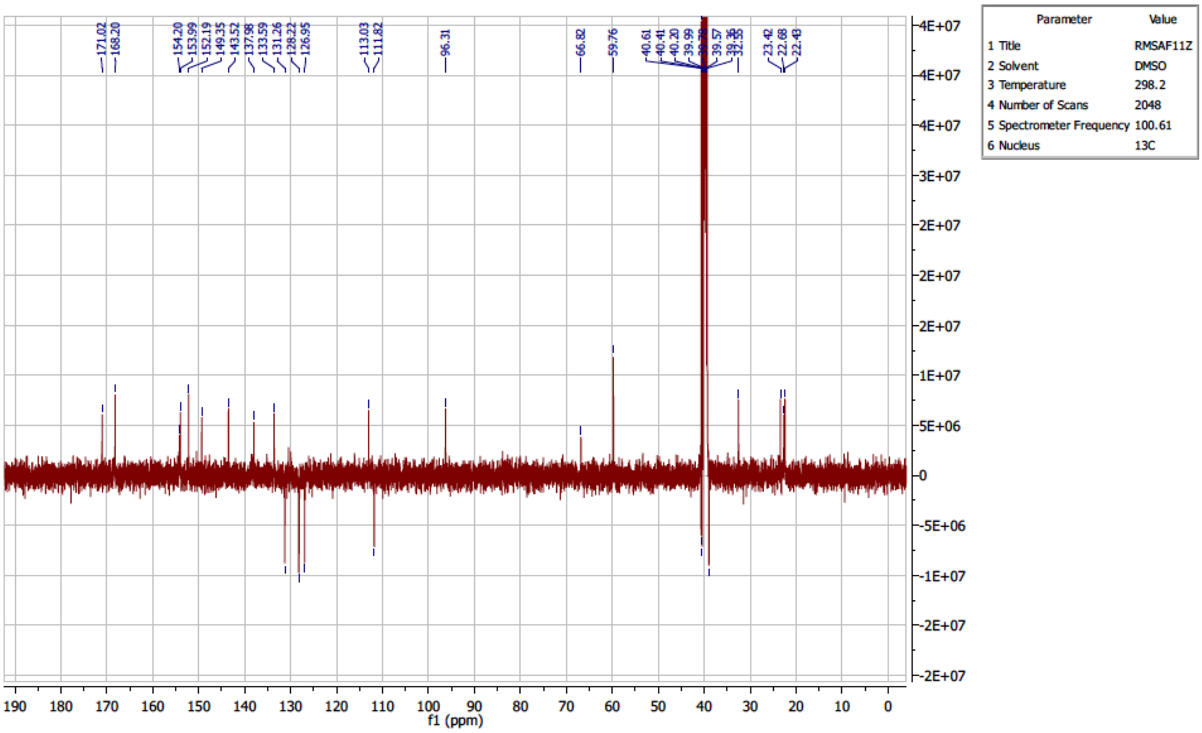

^{1}H NMR spectra of KT2k

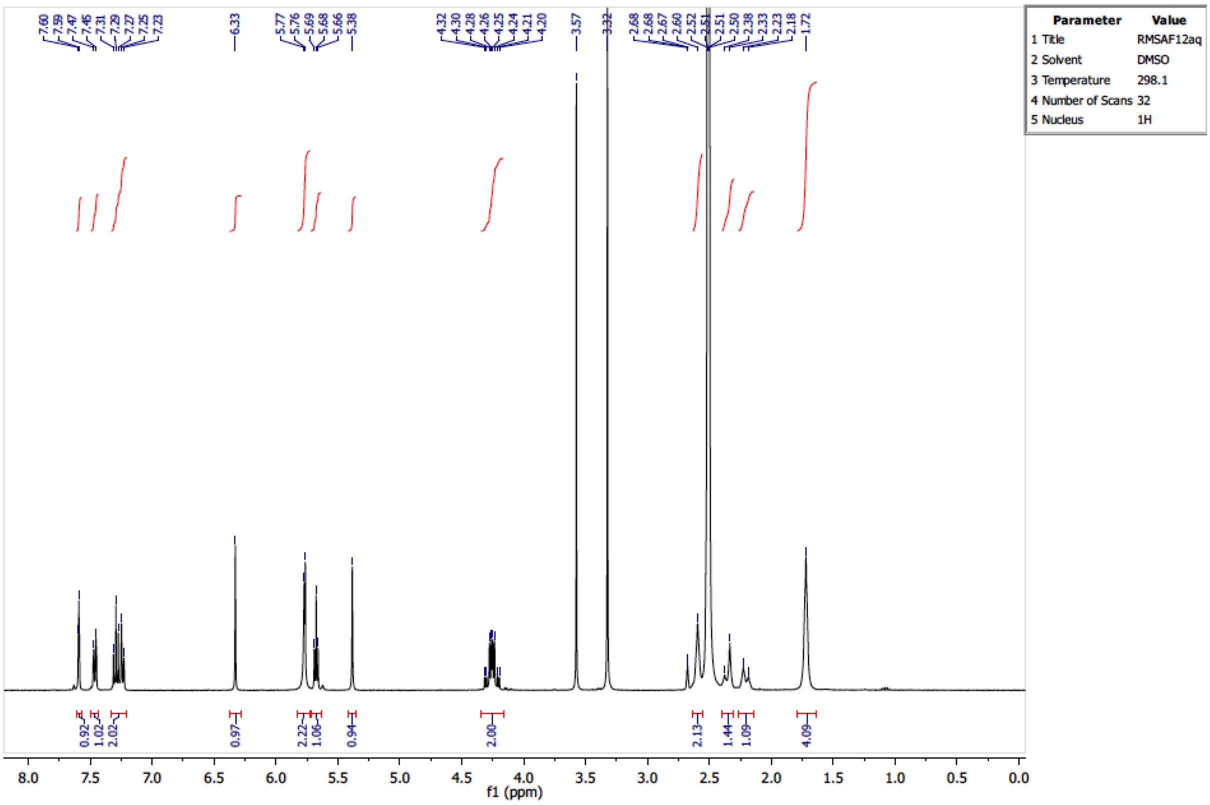

^{13}C NMR spectra of KT2k

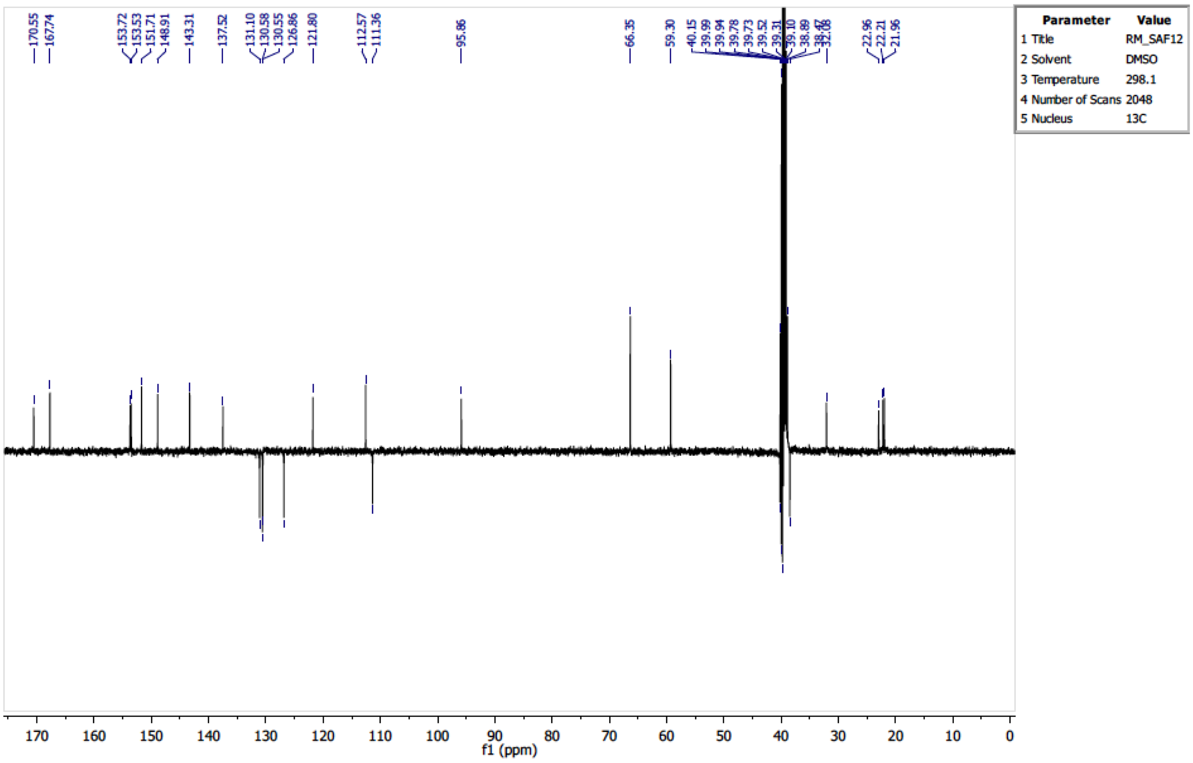

^{1}H NMR spectra of KT21

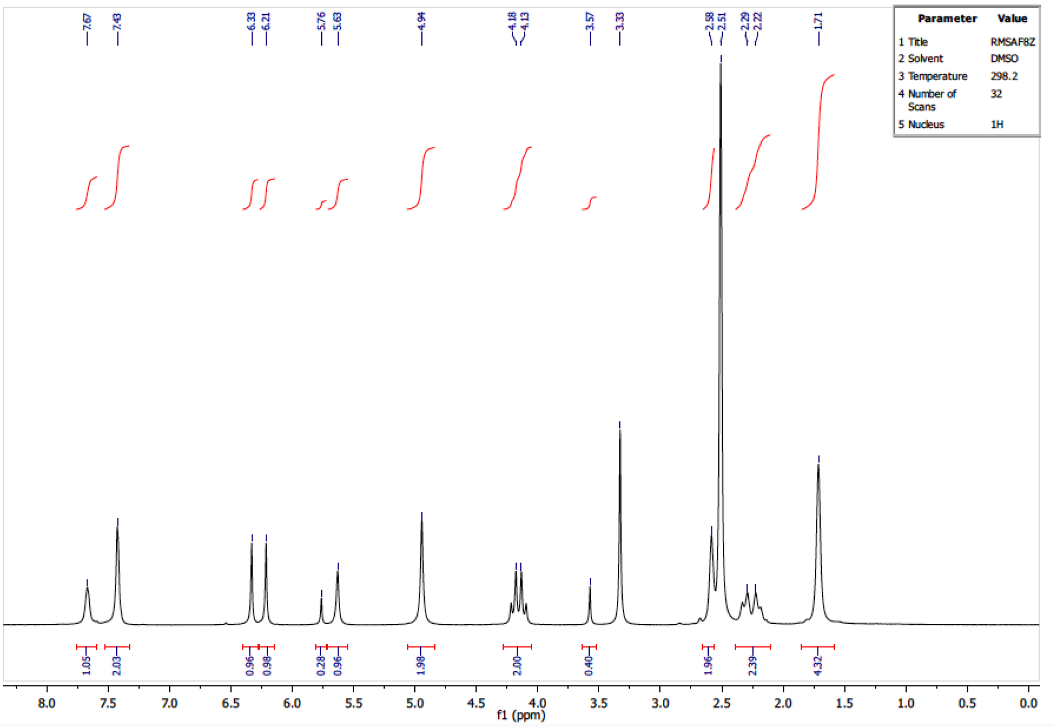

^{13}C NMR spectra of KT21

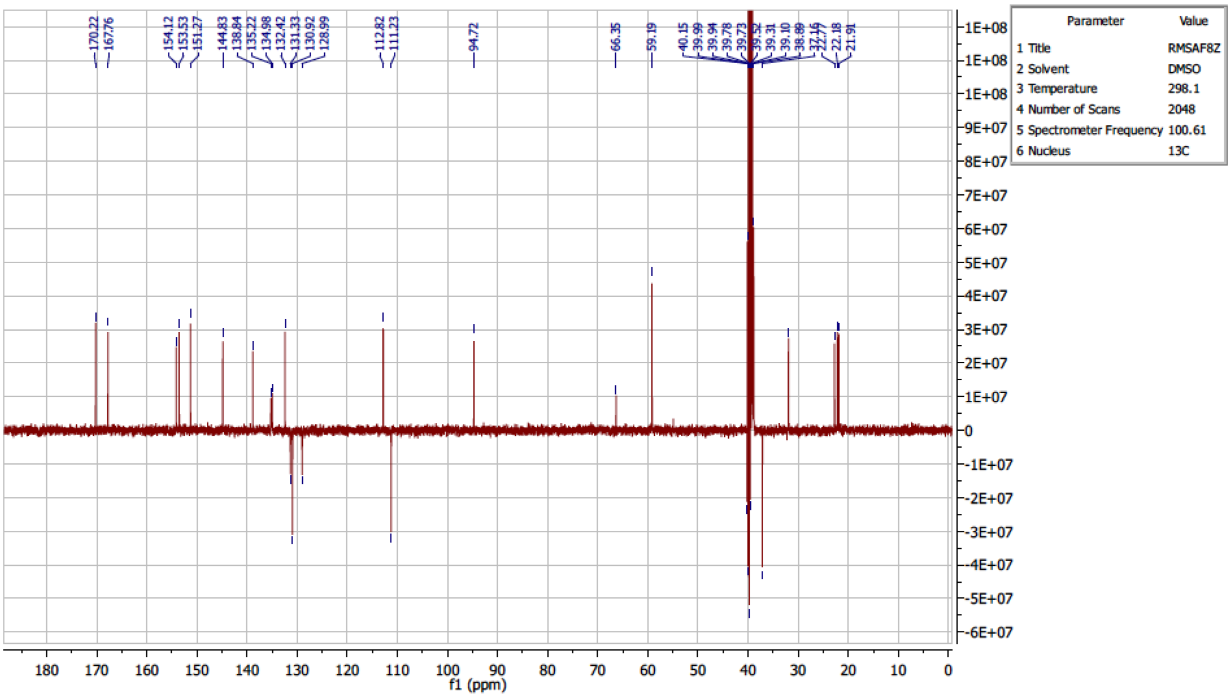

4. Binding mode and interaction of (*R*)**-KT2d** with hAChE

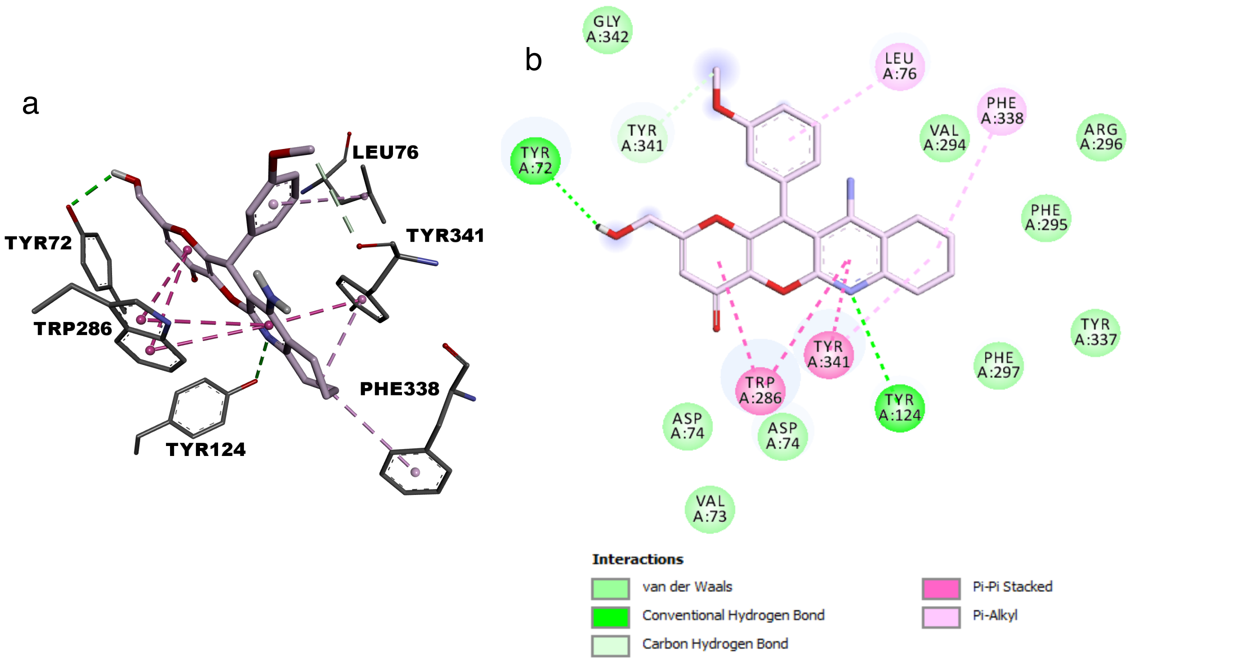

Figure S1. a) Illustration of (*R*)**-KT2d** binding mode in the hAChE active site. b) Schematic representation of different interactions of (*R*)**-KT2d** with hAChE.

5. Binding mode and interaction of (*S*)**-KT2d** with hAChE

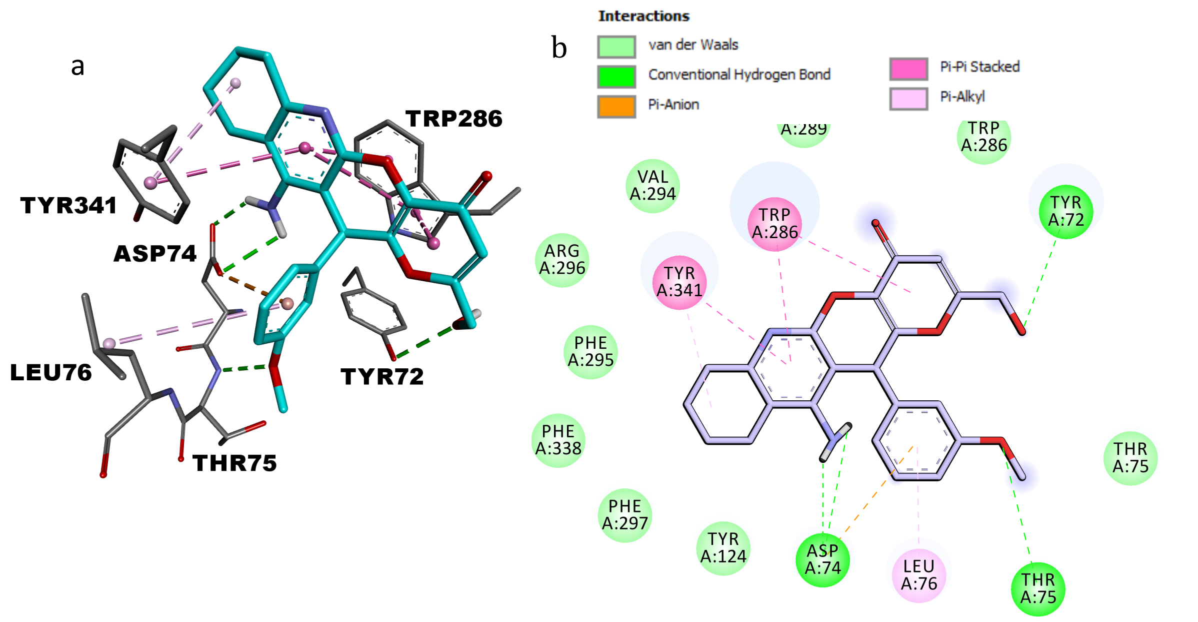

Figure S2. a) Interaction of compound (*S*)**-KT2d** docked to hAChE. b) Schematic 2D representation of different interactions of (*S*)**-KT2d** with hAChE.

6. ADME of compounds **KT2a-l**

Table S1. Physicochemical properties for compounds **KT2a-l** calculated using Qikprop

Molecule	MW	SASA	volume	donorHB	accptHB	QPlogPo/w	QPlogS
(*R*)-**2a**	376.411	633.779	1134.011	2.500	6.700	2.597	-4.898
(*S*)-**2a**	376.411	637.193	1135.699	2.500	6.700	2.559	-4.953
(*R*)-**2b**	390.438	666.494	1193.932	2.500	6.700	2.883	-5.436
(*S*)-**2b**	390.438	668.612	1191.170	2.500	6.700	2.818	-5.471
(*R*)-**2c**	406.437	650.972	1193.306	2.500	7.450	2.704	-4.806
(*S*)-**2c**	406.437	675.202	1217.891	2.500	7.450	2.760	-5.200
(*R*)-**2d**	406.437	653.798	1197.269	2.500	7.450	2.716	-4.852
(*S*)-**2d**	406.437	670.621	1207.608	2.500	7.450	2.662	-5.125
(*R*)-**2e**	406.437	644.920	1206.902	2.500	7.450	2.628	-5.181
(*S*)-**2e**	406.437	639.768	1201.580	2.500	7.450	2.676	-4.980
(*R*)-**2f**	394.401	637.680	1146.608	2.500	6.700	2.768	-5.089
(*S*)-**2f**	394.401	644.368	1145.814	2.500	6.700	2.727	-5.199
(*R*)-**2g**	394.401	674.008	1151.078	2.500	6.700	2.784	-5.281
(*S*)-**2g**	394.401	661.689	1147.369	2.500	6.700	2.817	-5.196
(*R*)-**2h**	394.401	650.415	1149.937	2.500	6.700	2.780	-5.248
(*S*)-**2h**	394.401	653.302	1144.867	2.500	6.700	2.819	-5.145
(*R*)-**2i**	410.856	640.922	1165.594	2.500	6.700	2.973	-5.223
(*S*)-**2i**	410.856	644.686	1167.693	2.500	6.700	2.940	-5.282
(*R*)-**2j**	410.856	642.934	1180.142	2.500	6.700	3.041	-5.657
(*S*)-**2j**	410.856	636.613	1175.537	2.500	6.700	3.076	-5.545
(*R*)-**2k**	455.307	661.093	1186.918	2.500	6.700	3.099	-5.720
(*S*)-**2k**	455.307	654.328	1186.205	2.500	6.700	3.145	-5.701
(*R*)-**2l**	445.301	663.395	1190.356	2.500	6.700	3.306	-5.555
(*S*)-**2l**	445.301	662.370	1193.015	2.500	6.700	3.298	-5.593

Molecule	QPPCaco	PSA	QPlogBB	metab	QPlogKhsa	% HOA	ROF	ROT
(*R*)-**2a**	317.779	99.351	-1.142	6	0.253	86.933	0	0
(*S*)-**2a**	274.343	99.389	-1.222	6	0.254	85.569	0	0
(*R*)-**2b**	314.925	99.373	-1.196	6	0.392	88.539	0	0
(*S*)-**2b**	272.802	99.320	-1.278	6	0.382	87.042	0	0
(*R*)-**2c**	395.554	103.946	-1.085	7	0.242	89.264	0	1
(*S*)-**2c**	314.721	104.534	-1.256	7	0.286	87.812	0	1
(*R*)-**2d**	402.739	107.282	-1.083	7	0.250	89.470	0	1
(*S*)-**2d**	290.300	107.713	-1.289	7	0.261	86.612	0	1

(*R*)-**2e**	272.181	108.118	-1.337	6	0.255	85.910	0	0
(*S*)-**2e**	345.641	107.402	-1.179	6	0.254	88.051	0	0
(*R*)-**2f**	326.242	98.806	-1.055	6	0.286	88.138	0	0
(*S*)-**2f**	293.239	98.826	-1.137	6	0.276	87.072	0	0
(*R*)-**2g**	277.508	99.419	-1.113	6	0.290	86.974	0	0
(*S*)-**2g**	329.063	99.150	-1.019	6	0.285	88.495	0	0
(*R*)-**2h**	280.217	99.475	-1.101	5	0.289	87.031	0	0
(*S*)-**2h**	345.136	98.858	-0.987	5	0.282	88.877	0	0
(*R*)-**2i**	351.523	99.715	-0.960	6	0.337	89.921	0	0
(*S*)-**2i**	307.367	99.699	-1.034	6	0.339	88.687	0	0
(*R*)-**2j**	277.685	99.449	-1.077	6	0.357	88.488	0	0
(*S*)-**2j**	336.188	99.037	-0.967	6	0.351	90.178	0	0
(*R*)-**2k**	275.290	99.511	-1.067	6	0.375	88.757	0	1
(*S*)-**2k**	320.623	99.305	-0.994	6	0.374	90.211	0	1
(*R*)-**2l**	415.486	98.670	-0.789	6	0.398	93.168	0	0
(*S*)-**2l**	393.289	98.175	-0.827	6	0.403	92.695	0	0

MW: Molecular weight of the molecule (130.0-725.0). SASA: Total Solvent Accessible Surface Area, in square angstroms, using a probe with a 1.4Å radius (limits 300.0-1000.0). volume: Total solvent-accessible volume, in cubic angstroms, using a probe with a 1.4 Å radius (limits 500.0-2000.0). donorHB: Estimated number of hydrogen bonds that would be accepted by the solute (limits: 2.0-20.0). accptHB: Estimated number of hydrogen bonds that would be donated by the solute (limits: 0.0-6.0). QPlogPo/w: Predicted octanol/water partition coefficient (limits -2.0-6.5). QPlogS: Predicted aqueous solubility. S, in mol/dm^3, is the concentration of the solute's saturated solution that is in equilibrium with crystalline solid (limits -6.5-0.5). QPPCaco: Predicted apparent Caco-2 cell permeability in nm/sec. Caco-2 cells is a model for the gut-blood barrier. QikProp predictions are for non-active transport. (< 25 poor, > 500 great). PSA: Van der Waals surface area of polar nitrogen and oxygen atoms (limits 7.0-200.0). QPlog BB: Predicted brain/blood partition coefficient (limits -3.0–1.2). metab: Number of likely metabolic reactions (limits 1-8). QPlogKhsa: Prediction of binding to human serum albumin (limits -1.5-1.5). HOA: Predicted qualitative Human Oral Absorption on 0 to 100% scale. ROF: Number of violations of Lipinski's Rule Of Five (Lipinski, C. A., Lombardo, F., Dominy, B. W., Feeney, P. J., "Experimental and computational approaches to estimate solubility and permeability in drug discovery and development settings", *Adv. Drug Delivery rev.* **2001**, 46, 3-26). (molecular weight < 500, QPlogPo/w < 5, number of hydrogen bond donor ≤ 5, number of hydrogen bond acceptors HB ≤ 10). ROT: Number of violations of Jorgensen's rule of three [(a) Duffy, E. M., Jorgensen,W. L., "Prediction of Properties from Simulations: Free Energies of Solvation in Hexadecane, Octanol, and Water", *J. Am. Chem. Soc.* 2000, 122, 2878-2888; (b) Jorgensen, W. L., Duffy, E. M., "Prediction of Drug Solubility from Monte Carlo Simulations", *Bioorg. Med. Chem. Lett.* **2000**, 10, 1155-1158](QPlogS> -5.7, QPCaco> 22 nm/s, number of primary metabolites < 7).

Theoretical calculations of the ADME (Absorption, Distribution, Metabolism and Excretion) properties of both enantiomers of tacrine derivatives **KT2a-l** were accomplished using the QikProp module of Schrodinger suite (QikProp, version 3.8, Schrodinger, LLC, New York, NY, 2013) running in normal mode, for assessing the druggability. Drug kinetics and exposure of tissues to drug influences the pharmacological activity and the performance of a drug, which is ultimately determined by its ADME properties. Nearly 45 physically significant descriptors and pharmacologically relevant properties were predicted and investigated (Table S1). The results suggest that the compounds do not violate Lipinski's rule (0). Almost all calculated descriptors and properties except the estimated number of hydrogen bonds that would be donated by the solute (acceptHB) are within the expected thresholds. Aqueous solubility (QPlogS) of organic molecules plays a key impact on many ADME-related properties. All compounds showed solubility values within the limits. The partition coefficient (QPlogPo/w), critical for estimation of absorption within the body, ranged between -2.56 and 3.30 (Table S1). Among all the properties, the predicted value for the Blood Brain Barrier (BBB) penetration (QPlogBB: acceptable range −3.0 to 1.2) is a very important parameter, indicating the ability of the molecule to pass through the blood brain barrier, which is mandatory for Alzheimer's treatment. The predicted logBB values for compounds **KT2a-l** (Table S1) are in the optimum penetrate range. Literature survey suggests that Polar Surface Area (PSA) is a measure of a molecule's hydrogen bonding capacity and its value should not exceed a certain limit if the compound is intended to be CNS active. Molecules with PSA <100 **Å**^{2} are more likely to penetrate the BBB and the most active CNS drugs have PSA lower than 70 **Å**^{2}. The values of PSA for compounds KT**2a-l** are in the range from 97.17 to 108.12 A^2 and they are within the range given by QikProp. The human percentage oral absorption for the compounds is high (85.60 %-93.17 %) (Table S1). Other physicochemical descriptors obtained by QikProp (Table S1) are within the acceptable scope for human use, thus making these derivatives as suitable drug candidates and possible CNS drug.

7. References

1. Baghbanian SM, Rezaei N, Tashakkorian H. Nanozeolite clinoptilolite as a highly efficient heterogeneous catalyst for the synthesis of various 2-amino-4H-chromene derivatives in aqueous media. *Green Chem.* 15(12), 3446–3458 (2013).
2. Piao M-Z, Imafuku K. Convenient synthesis of amino-substituted pyranopyranones. *Tetrahedron Lett.* 38(30), 5301–5302 (1997).
3. Kataev EA, Reddy MR, Reddy GN, Reddy VH, Reddy CS, Reddy BVS. Supramolecular catalysis by β-cyclodextrin for the synthesis of kojic acid derivatives in water. *New J. Chem.* 40(2), 1693–1697 (2016).
4. Banitaba SH, Safari J, Khalili SD. Ultrasound promoted one-pot synthesis of 2-amino-4,8-dihydropyrano[3,2-b]pyran-3-carbonitrile scaffolds in aqueous media: A complementary 'green chemistry' tool to organic synthesis. *Ultrason. Sonochem.* 20(1), 401–407 (2013).
5. Meng X-X, Du B-X, Zhao B, Li Y-L, Chen C-F. An efficient three-component synthesis of amino-substituted pyrano[3,2-b] pyranones. *J. Chem. Res.* 37(10), 638–641 (2013).
